# Identifying cases of chronic pain using health administrative data: A validation study

**DOI:** 10.1080/24740527.2020.1820857

**Published:** 2020-12-03

**Authors:** Heather E. Foley, John C. Knight, Michelle Ploughman, Shabnam Asghari, Rick Audas

**Affiliations:** a Division of Community Health and Humanities, Faculty of Medicine, Memorial University of Newfoundland, St. John’s, Newfoundland and Labrador, Canada; b Primary Health Care Research Unit, Faculty of Medicine, Memorial University of Newfoundland, St. John’s, Newfoundland and Labrador, Canada; c Physical Medicine & Rehabilitation, Faculty of Medicine, Memorial University of Newfoundland, St. John’s, Newfoundland and Labrador, Canada; d Discipline of Family Medicine, Faculty of Medicine, Memorial University of Newfoundland, St. John’s, Newfoundland and Labrador, Canada

**Keywords:** chronic pain, validation, health administrative data, algorithm, population-based, electronic medical records data, case ascertainment

## Abstract

**Background:**

Most prevalence estimates of chronic pain are derived from surveys and vary widely, both globally (2%–54%) and in Canada (6.5%–44%). Health administrative data are increasingly used for chronic disease surveillance, but their validity as a source to ascertain chronic pain cases is understudied.

**Aim:**

The aim of this study was to derive and validate an algorithm to identify cases of chronic pain as a single chronic disease using provincial health administrative data.

**Methods:**

A reference standard was developed and applied to the electronic medical records data of a Newfoundland and Labrador general population sample participating in the Canadian Primary Care Sentinel Surveillance Network. Chronic pain algorithms were created from the administrative data of patient populations with chronic pain, and their classification performance was compared to that of the reference standard via statistical tests of selection accuracy.

**Results:**

The most performant algorithm for chronic pain case ascertainment from the Medical Care Plan Fee-for-Service Physicians Claims File was one anesthesiology encounter ever recording a chronic pain clinic procedure code OR five physician encounter dates recording any pain-related diagnostic code in 5 years with more than 183 days separating at least two encounters. The algorithm demonstrated 0.703 (95% confidence interval [CI], 0.685–0.722) sensitivity, 0.668 (95% CI, 0.657–0.678) specificity, and 0.408 (95% CI, 0.393–0.423) positive predictive value. The chronic pain algorithm selected 37.6% of a Newfoundland and Labrador provincial cohort.

**Conclusions:**

A health administrative data algorithm was derived and validated to identify chronic pain cases and estimate disease burden in residents attending fee-for-service physician encounters in Newfoundland and Labrador.

## RÉSUMÉ

**Contexte**: La plupart des estimations de prévalence de la douleur chronique sont tirées d’enquêtes et varient considérablement, à la fois dans le monde (2 % -54 %) et au Canada (6,5 % - 44 %). Les données administratives sur la santé utilisées pour la surveillance des maladies chroniques, mais leur validité comme source pour déterminer les cas de douleur chronique est sous-étudiée.

**Objectif**: Le but de cette étude était de dériver et de valider un algorithme pour répertorier les cas de douleur chronique comme une seule maladie chronique en utilisant les données administratives provinciales sur la santé.

**Méthodes**: Une norme de référence a été élaborée et appliquée aux données des dossiers médicaux électroniques d’un échantillon de la population générale de Terre-Neuve-et-Labrador participant au Réseau canadien de surveillance sentinelle en soins primaires. Des algorithmes de douleur chronique ont été créés à partir des données administratives de populations de patients souffrant de douleur chronique et leur rendement en matière de classification a été comparé à celui de la norme de référence par le biais de tests statistiques sur la précision de sélection.

**Résultats**: L’algorithme le plus performant pour la détermination des cas de douleur chronique à partir du Registre des paiements des soins médicaux rémunérés à l’acte était une seule consultation en anesthésiologie au cours de laquelle un code de procédure d’intervention clinique en matière de douleur chronique était enregistré OU cinq consultations médicales en cinq ans au cours desquelles était enregistré tout code de diagnostic lié à la douleur, avec une période de plus de 183 jours entre au moins deux consultations.

L’algorithme a démontré une sensibilité de 0,703 (intervalle de confiance [IC] à 95 %, 0,685 à 0,722), une spécificité de 0,668 (IC 95 %, 0,657-0,678) et une valeur prédictive positive de 0,408 (IC 95 %, 0,393-0,423). L’algorithme de la douleur chronique a sélectionné 37,6 % d’une cohorte provinciale de Terre-Neuve-et-Labrador.

**Conclusions**: Un algorithme de données administratives sur la santé a été dérivé et validé pour répertorier les cas de douleur et estimer le fardeau de la maladie chez les résidents ayant consulté un médecin rémunéré à l’acte à Terre-Neuve et Labrador.

## Introduction

Chronic pain is a pervasive and challenging public health issue.^1–5^ Globally, prevalence estimates range drastically from 2% to 54%,^2,4,6–11^ with similar variability reported in Canada (6.5%–44%).^3,12–21^ Such variability in prevalence creates uncertainty when planning for present and future health care needs. Annual costs related to chronic pain in Canada are expected to exceed over US$10 billion by 2025.^19,22,23^ In Canada, most chronic pain prevalence estimates were derived from national or regional surveys.^3,12,15–20^ Although surveys provide descriptive information, they are expensive and labor intensive.^24^ Another easily accessible and low-cost method to obtain prevalence estimates is to use algorithms applied to health administrative data collected by provinces in Canada.^25^ There is a paucity of studies examining whether cases of chronic pain, a complex and multifaceted condition, could be extracted from administrative data, with specific chronic pain conditions often being the focus.^26–31^ However, most queries from a policy perspective center on chronic pain as a single chronic disease.^32^ If accurate and valid, using health administrative data as an information source will enable a rapid and efficient method to obtain important epidemiological, health planning, and policy data on this significant chronic condition.

Each province and territory in Canada administers universal health plans that cover most hospital and physician services to nearly all of their residents.^33^ Despite only capturing information obtained through physician and hospital encounters, the health administrative data generated are used to extract annual population-based estimates on distribution, trends and direct health care costs of various medical conditions in Canada through validated algorithms.^34^ Previous studies on chronic pain that used administrative data ascertained cases through convenience samples,^35^ surveys,^36^ code sets not previously validated,^4,37^ or validated algorithms for specific pain conditions,^26–29^ such as low back pain.^30^ One study successfully derived a chronic pain case definition for electronic medical record data,^11^ but the clinical information utilized (in an American health care setting) is not universally collected and is not available in Canadian administrative data.^38,39^

The growing dependence on administrative data for chronic disease surveillance emphasizes the importance of using valid algorithms for case ascertainment.^25^ The challenge of using health administrative data sets is that record-level data are not collected for research purposes and may have significant data entry errors.^25,40^ This is exacerbated by chronic pain often being considered a symptom of another trauma or disease process with no objective diagnostic “gold standard” to use for validation,^1,4,11,41,42^ unlike other chronic diseases with standard objective diagnostic tests such as diabetes,^43,44^ multiple sclerosis,^40^ and rheumatoid arthritis.^45^ Applying standardized methodology to create, validate, and report administrative data algorithms that identify cases of chronic pain as “a single disease entity”^46(p1682)^ advances the utility of the information obtained and examined by researchers, clinicians, and health policymakers.^25^

An administrative data algorithm is a combination of diagnostic and procedural code patterns (known as spatial frequency) together with encounter frequency patterns (known as temporal frequency).^25,47^ It operates similar to diagnostic testing in medical practice.^25,47^ A chronic pain algorithm must include spatial and temporal frequency criteria that align with accepted practice in the diagnosis of chronic pain.^25^ A standardized set of diagnostic and/or procedural codes is required to identify chronic pain–related conditions and treatments in administrative data.^25^ Pain extending beyond 3 months post onset, or 6 months for the purposes of research, as defined by The International Association for the Study of Pain is the required temporal benchmark for chronic pain case ascertainment.^48^ A review of 11 studies in the field revealed 11 different chronic pain definitions and/or code sets used in research.^2–4,7–10,15,20,37,49^ Currently, there is no consistency in chronic pain research regarding appropriate spatial and temporal frequency.

The aim of the present study was to determine whether Canadian health administrative data would provide valid information on cases of chronic pain in the context of a single disease. The study sought to achieve this by using administrative data collected in one Canadian province, Newfoundland and Labrador (NL), to develop an algorithm with the appropriate spatial and temporal criteria. Validity and reliability were examined against an electronic medical record database audit. This study marks the first step in addressing the long-term goal of compiling detailed statistics on the chronic pain condition in the Canadian context, which can be used to inform policy around health service provision for this high-needs population.

## Materials and Methods

The Health Research Ethics Board (HREB) of the Health Research Ethics Authority of Newfoundland and Labrador provided full approval of the study protocol (HREB Reference #13.157). The Secondary Uses Committee of the NL Center for Health Information and the Research Proposals Approval Committee of the Eastern Regional Health Authority also reviewed and approved the study protocol following HREB approval.

### Setting

The Canadian Primary Care Sentinel Surveillance Network (CPCSSN)-NL data were used for algorithm validation. The CPCSSN is a clinical data source comprised of information retrieved directly from the electronic medical records of patients attending participating primary care practices across Canada.^50^ In February 2013, 45 physicians (approximately 9% of the NL registered primary care physicians) ^51^ practicing in 8 primary care clinics in mainly urban NL was annually contributing de-identified electronic medical records data on just over 35,000 patients of all ages (approximately 7% of the NL population)^52^ to the Canadian Primary Care Sentinel Surveillance Network-NL dataset.^53^

The primary care physicians participating in the CPCSSN database provided written consent on behalf of their patients to have their patient electronic medical record data regularly transferred to the CPCSSN, which follows strict and secure privacy protocols when using the de-identified data from patients’ electronic medical records. Data sharing and confidentiality agreements were put in place. The participating primary care physicians provided written information (posters and pamphlets in their offices) to patients about the CPCSSN and how their data will be used and that they had the option to opt out of data collection at any time. The ethics approval obtained for the CPCSSN project in NL included a waiver of explicit patient consent because of the infeasibility of obtaining individual consent for the large geographical population involved in the project and because only secondary data analysis of preexisting de-identified data was performed. Patients’ consent to participate in the CPCSSN database and for their de-identified information in the electronic medical record to be used for research purposes, including data linkages, was thus implied.^54,55^

The CPCSSN data tables containing medical record information utilized for the purposes of this study included the Encounter, Encounter Diagnosis, Health Conditions, Medication, Patient Demographics, and Provider tables. These tables contained clinical information extracted directly from each entry in the medical record and included raw text, diagnostic codes, Anatomical Therapeutic Chemical Classification codes (medication codes), procedures performed, and relevant dates (e.g., dates of encounters and medication start–stop dates) as entered by the attending primary care physician.^54^ The World Health Organization maintains and updates a standardized system of numeric or alphanumeric codes to classify medical diagnoses called the *International Classification of Diseases* (ICD), and the CPCSSN utilizes three- to five-digit codes from the ninth revision of the ICD (ICD-9).^54,56^ Clinical data are organized via the patient’s unique health insurance number and are de-identified prior to data transfer to CPCSSN.^50^ The CPCSSN data undergo rigorous quality control procedures; the CPCSSN was previously determined to be a valid data source to study eight chronic diseases^57^ and a valid proxy (77.5%–97.2% sensitivity and 93.1%–99.4% specificity) to manual review of electronic chart raw data for validation studies.^58^

### Reference Standard Cohort and Reference Standard

The reference standard cohort was comprised of primary care patients of all ages who met the inclusion criteria of implied consent to participate in the CPCSSN-NL since December 31, 2009, or earlier and a minimum of 2 years of electronic medical record data for analysis. Because the CPCSSN-NL data have only been collected since 2005,^50^ the data range from January 1, 2006, to December 31, 2011, was extracted for this cohort.

The presence of chronic pain in the reference standard cohort was determined using both spatial and temporal benchmarks that align with a chronic pain definition. The temporal benchmark was defined as persistent or recurrent pain lasting longer than 6 months.^42,48^ A comprehensive search of all sources of clinical information for evidence of assessment/treatment of pain-/chronic pain–related conditions was performed by one of the authors with clinical expertise in chronic pain (H.F.). A combination of ICD-9 diagnostic codes, Anatomical Therapeutic Chemical Classification codes, medication start–stop dates, raw and cleaned textual data, and encounter frequency from the CPCSSN data served as the CPCSSN-NL reference standard for chronic pain. The spatial benchmark for the reference standard was informed by published literature,^4,11,26,28–30,37,41,59–65^ consultation with chronic pain experts (H.F., E.T., and J.F.) and a pharmacy expert (C.D.), and codes/text utilized in the CPCSSN-NL data. Patients in the reference standard cohort were classified as having chronic pain if any one of the following CPCSSN-NL data criteria was met in the cumulative patient electronic medical record data up to December 31, 2011: (1) a single encounter date recording (ICD-9 diagnostic codes 338.0, 338.2,^a^ [a]^a^*International Classification of Disease*, Ninth Revision diagnostic codes of 338.0 and 338.2 are not utilized in Canadian Primary Care Sentinel Surveillance Network–Newfoundland and Labrador data but are included in this article for completeness. or 338.4) OR (text with “chronic” and “pain” in the same text entry not necessarily following each other); OR (2) receipt of at least 90 days of opioid medication used almost exclusively for pain (Table S1, Supplementary file 1) in the CPCSSN-NL study period; OR (3) four or more encounter dates recording (any ICD-9 pain-related diagnostic code; Table S2, Supplementary file 1) OR (text with “pain”) within a 2-year period with more than 183 days separating at least two pain-related encounter dates.

### Administrative Data Sources

Two administrative data sources were used for the chronic pain algorithms: (1) the Provincial Discharge Abstract Database (NL Discharge Abstract Data), which is the NL component of the Canadian Institute of Health Information national Discharge Abstracts Database, containing information on all separations from acute health care facilities in NL, including admission date and up to 16 diagnostic codes, and (2) Medical Care Plan (MCP) Fee-for-Service Physicians Claims File (MCP Claims File) containing information, including one diagnostic code and one provincial billing code, on all claims for health services provided by fee-for-service physicians in NL. All data are organized by each NL resident’s unique health insurance number.^38,66^

All NL Discharge Abstract and MCP Claims File data are used for research and surveillance of multiple injuries and disease states.^34^ Rigorous quality control procedures are applied to the NL Discharge Abstract data on an annual basis, and MCP Claims File data are considered complete due to their collection for service remuneration.^53,67,68^ The MCP Beneficiary File was used to obtain demographic and benefits eligibility information, including age, sex, rural/urban location of residence, and health authority region of residence. All required record-level data from January 1, 1999, to March 31, 2010, were obtained from these data sets.

The NL Discharge Abstract Data used five-digit ICD-9 codes up to March 31, 2001, and six-digit *International Classification of Disease*–10th Revision (Canadian) (ICD-10-CA) codes from April 1, 2001, onwards. The MCP Claims File data used three-digit ICD-9 codes throughout the data study period. Although the 11th revision of the ICD contains specific classifications of chronic pain conditions,^42^ the ICD-9 and ICD-10-CA do not.^56^ To determine the spatial benchmark and account for the many proxies used by clinicians and researchers for pain-related diagnoses,^4,11,37,41^ previous studies and consultation with pain experts (H.F., E.T., and J.F.) were used to select the pain-related ICD-9 and ICD-10-CA diagnostic codes (Table S3, Supplementary file 1) searched in the NL administrative data.^4,11,26,28–30,37,41,59–64^ Chronic pain–related provincial procedure billing codes (Table S4, Supplementary file 1) searched in the MCP Claims File were reserved for medical assessment and treatment of people with chronic pain carried out by anesthesiologists in organized hospital pain clinics.^69^

### Administrative Data Algorithms

Convenience samples of known chronic pain cases were obtained to develop and sensitivity test preliminary chronic pain algorithms. Inclusion criteria for the pain patient populations were (1) attending an interdisciplinary chronic pain rehabilitation program from 2006 to 2011, (2) attending an interdisciplinary chronic pain rehabilitation program from 1999 to 2005, (3) being on the waitlist to attend an interdisciplinary chronic pain rehabilitation program on September 1, 2012, or (4) being prescribed and dispensed any opioid medication used almost exclusively for pain (Table S1, Supplementary file 1) during the period from 1999 to 2011 as a subsidized patient of the NL Prescription Drug Program. The interdisciplinary chronic pain rehabilitation program is located in St. John’s, NL, and is known as the Center for Pain and Disability Management.^70^ The NL Prescription Drug Program provides financial assistance for eligible prescription medications to qualified seniors and low-income individuals/families.^71^

Because the health administrative data analyzed were part of routine data collection and normal operations of the NL Center for Health Information, NL Prescription Drug Plan, and the Eastern Regional Health Authority and the data was then de-identified, individual patient and/or NL resident consent was not required.

For the algorithm development step, MCP Claims File and NL Discharge Abstract Data for the pain patient population attending the interdisciplinary chronic pain rehabilitation program from 2006 to 2011 were searched for the presence of pain-related diagnostic and procedure codes (spatial benchmarks). Encounter and hospitalization dates associated with pain-related diagnostic codes were searched for the presence of the 6-month temporal benchmark. Preliminary algorithms were created by combining the presence of (1) up to five dates of encounters and/or hospitalizations with any physician recording any pain-related diagnostic code (Table S3, Supplementary file 1) in either the MCP Claims File or NL Discharge Abstract Data, (2) one or more encounters with a medical specialist recording any pain-related diagnostic code (Table S3, Supplementary file 1) in either the MCP Claims File or NL Discharge Abstract Data, (3) more than 183 days separating at least two encounter dates with a physician recording any pain-related diagnostic code in the MCP Claims File or the NL Discharge Abstract Data, and (4) chronic pain–related physician procedure billing codes (Table S4, Supplementary file 1) in the MCP Claims File. Initially, the algorithms were observed for all available years of the data (1999–2010). The algorithms were then observed for specified time windows to maximize potential chronic disease surveillance utility. A previous study identified up to 7 years as the optimal clearance period for recurrent low back pain^72^; therefore, the time window of between 1 and 7 years was chosen to observe required algorithm spatial and temporal benchmarks.

For the preliminary algorithm sensitivity testing step, the algorithms were tested for sensitivity on the administrative data of the four pain patient population groups.

**Figure 1.**
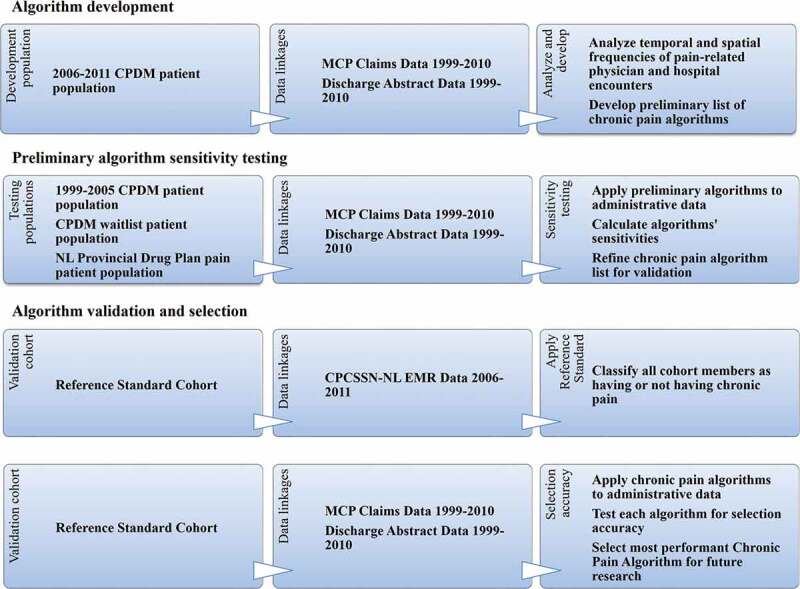
Summary of study methodology and associated data flow. ^a^The NL Prescription Drug Plan is a financial assistance program covering eligible prescription medications to qualified seniors and low-income individuals/families. Patients selected were prescribed and dispensed opioid medication used almost exclusively for pain (Table S1, Supplementary file 1) during the period from 1999 to 2011 as a subsidized patient of the NL Prescription Drug Program. CPDM = Center for Pain and Disability Management (an interdisciplinary chronic pain rehabilitation program); MCP = Medical Care Plan; NL = Newfoundland and Labrador; CPCSSN = Canadian Primary Care Sentinel Surveillance Network; EMR = electronic medical record

For the algorithm validation and selection step, a refined list of algorithms was selected, applied to the reference standard cohort administrative data, and rigorously tested for validity via multiple statistical tests of selection accuracy comparing administrative data case ascertainment to that of the reference standard. In all steps, the administrative data algorithm classified pain patient population group or validation cohort members as having chronic pain if the algorithm criteria were met at any time in the administrative data period (1999–2010). Using the entire data period accommodated both the nature of chronic pain as having no cure and the uncertain timing of diagnosis due to the lack of a standard objective diagnostic test. Figure 1 summarizes the methodology and associated data flow.

### Algorithm Application to a Provincial Cohort

Once the most performant algorithm to identify chronic pain cases from administrative data was selected, it was applied to the administrative data of a provincial cohort of NL residents. All residents identified as eligible for MCP benefits (approximately 98% of the total NL population) in the MCP Beneficiary File for any fiscal year between 2003 and 2010 were included in the provincial cohort, of which 99.6% had linkages to the MCP Claims File (fee-for-service physician visits) and 65.3% had linkages to the NL Discharge Abstract Data (acute care hospitalizations).

### Data Linkage

The CPCSSN-NL data, NL Discharge Abstract Data, and MCP Claims File data were obtained from the NL Center for Health Information.^66^ The CPCSSN-NL data were linked to the reference standard cohort via the unique provincial health insurance (MCP) numbers. Record-level data from the MCP Claims File and NL Discharge Abstract Data were linked to the reference standard cohort, the interdisciplinary chronic pain rehabilitation program patient populations, the NL Prescription Drug Plan pain patient population, and the provincial cohort via MCP numbers. Analysts at the NL Center for Health Information performed all data extraction, linkage, cleaning, and de-identification prior to provision of the linked data sets to the research team for analysis.

### Statistical Analysis

Distribution of chronic pain cases as defined by the reference standard in the reference standard cohort were described and compared to those not identified as having chronic pain through a *t* test for mean age and chi-square tests for proportions (statistical significance defined by *P* < 0.05). Preliminary algorithm sensitivity was calculated in each pain patient population by dividing algorithm-selected cases by the total corresponding pain patient population.

For algorithm validation and selection, the chronic pain algorithms were applied to the administrative data of the reference standard cohort, and algorithm classification performance was compared to that of the reference standard. There are complexities inherent to validating chronic disease administrative data algorithms, including (1) multiple required health care provider encounters to deem the disease chronic; (2) multiple codes entered for the same medical issue as the provider works to “rule out” other conditions to arrive at the best diagnosis; (3) varying prevalence of the chronic disease in a population based on age, sociodemographics, and geographic location (an indicator of health service availability); and (4) varying severity of disease according to individuals.^25^ A broad range of statistical tests for accuracy and their 95% confidence intervals (CIs) were calculated for each proposed administrative data algorithm using the classic 2 × 2 table to adequately account for these complexities and to sufficiently illustrate algorithm performance.^25,73^

Sensitivity and specificity assessed case ascertainment utility, and positive predictive value, negative predictive value, likelihood ratio positive, likelihood ratio negative, and diagnostic odds ratio assessed selection accuracy.^25,47,74,75^ The kappa agreements between each administrative data algorithm and the CPCSSN reference standard were calculated using the classic 2 × 2 table.^75–77^ The area under the receiver operating characteristic curve, also a selection accuracy test, for each proposed algorithm was obtained.

To optimize algorithm functionality in assessing the disease burden of chronic pain, the research team sought to maximize case selection while minimizing false positives. The most performant algorithm was chosen based on the balance between sensitivity and specificity while maximizing positive predictive value,^43,47,78^ with the goal of each being greater than 0.70.^21^ A plot of calculated sensitivity and specificity values for each algorithm was made and the intersection of the plot lines assisted in choosing the most performant algorithm. Once the selected most performant chronic pain algorithm was applied to the reference standard cohort administrative data, identified false-positive and false-negative cases were reviewed in further detail. Finally, the most performant chronic pain algorithm was applied to validation cohort strata for age (14 years and under, 15–24 years, 25–34 years, 35–44 years, 45–54 years, 55–64 years, 65–79 years, and 80 years and over) and sex (male and female), and its selection accuracy at each stratum was assessed for potential differences in performance. SPSS v24 and Excel 2013 were used for statistical analysis.

## Results

### Reference Standard

**Figure 2.**
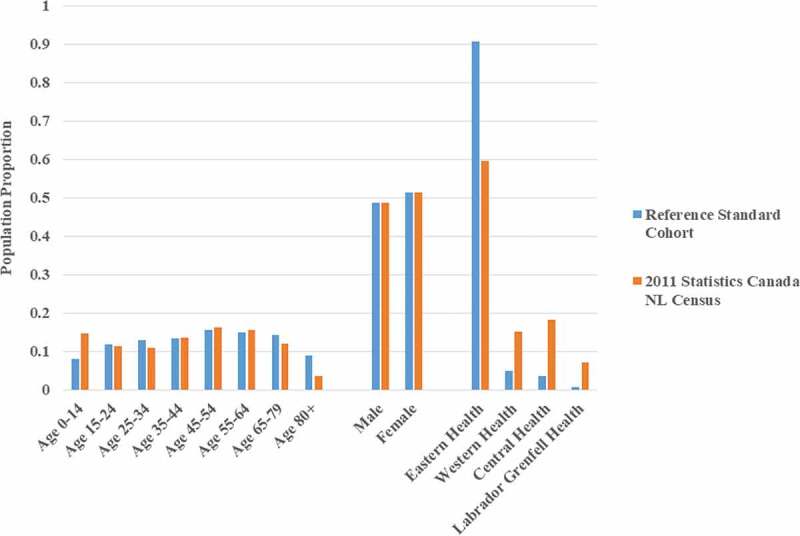
2011 Demographics of the reference standard cohort versus the Newfoundland and Labrador general population. Proportions of the reference standard cohort and the Statistics Canada 2011 census-reported Newfoundland and Labrador population in each age, sex, and Regional Health Authority stratum are compared. Eastern, Western, Central, and Labrador–Grenfell are the four Regional Health Authorities in Newfoundland and Labrador. NL = Newfoundland and Labrador

Compared to the Statistics Canada 2011 census-reported NL general population (Figure 2),^79–83^ the 2011 demographics of the reference standard cohort had similar sex distribution but a higher median age (48.0 years vs. 44.0 years). The reference standard cohort had an overrepresentation of people aged 65 and over and underrepresentation of people aged 14 and under. There was also a higher percentage of people in the reference standard cohort residing in the Eastern Regional Health Authority (mostly urban) catchment area. The Eastern Regional Health Authority is one of four located in NL.

Table 1 details the distribution of chronic pain cases in the reference standard cohort. Chronic pain prevalence as defined by the reference standard was 24.6%, of which 58.8% were identified as female and 54.2% were aged 55 or older. Mean age was significantly higher at 55.5 years (standard deviation (SD) = 19.1 years) in the chronic pain group versus 44.1 years (SD = 22.9 years) in the no chronic pain group (*P* < 0.001).

**Table 1.** 2011 Demographics of chronic pain group vs. no chronic pain group in reference standard cohort.^a^

	Chronic pain group *N* = 2386	No chronic pain group *N* = 7329	
	*N* (% of group)	*N* (% of group)	*P* value
Age group			
0–14	32 (1.3)	755 (10.3)	<0.001
15–24	133 (5.6)	1005 (13.7)	<0.001
25–34	206 (8.6)	1039 (14.2)	<0.001
35–44	288 (12.1)	1019 (13.9)	0.0248
45–54	435 (18.2)	1081 (14.7)	<0.001
55–64	521 (21.8)	934 (12.7)	<0.001
65–79	500 (21.0)	892 (12.2)	<0.001
80+	271 (11.4)	604 (8.2)	<0.001
Sex			
Male	984 (41.2)	3744 (51.1)	<0.001
Female	1402 (58.8)	3585 (48.9)	<0.001
Regional Health Authority			
Eastern	2262 (94.8)	6548 (89.3)	<0.001
Central	34 (1.4)	321 (4.4)	<0.001
Western	84 (3.5)	390 (5.3)	<0.001
Labrador–Grenfell	6 (0.3)	67 (0.9)	0.0018
Pain conditions^b^			
Neuropathic pain	766 (32.1)	531 (7.2)	<0.001
Musculoskeletal conditions and arthritis	1715 (71.9)	1853 (25.3)	<0.001
Musculoskeletal trauma	864 (36.2)	883 (12.0)	<0.001
Neck and back pain	1546 (64.8)	1412 (19.3)	<0.001
Bone disorders	427 (17.9)	353 (4.8)	<0.001
Headaches	700 (29.3)	631 (8.6)	<0.001
Other conditions associated with chronic pain	1567 (65.7)	1729 (23.6)	<0.001
Central pain syndrome, chronic pain, or chronic pain syndrome	98 (4.1)	0	<0.001

^a^Chi-square tests were used to determine significance of difference between group proportions. Statistical significance was defined by *P* < 0.05. Difference between the proportions of the chronic pain group and the no chronic pain group in all strata were considered significant. ^b^Inclusion in the pain condition group was defined as an individual having ≥1 encounter for any condition in the pain condition diagnostic group (Table S2, Supplementary file 1) in the Canadian Primary Care Sentinel Surveillance Network–Newfoundland and Labrador electronic medical record data at any time from January 1, 2006, to December 31, 2011. A cohort member could be counted as a case in more than one pain condition diagnostic group.

### Administrative Data Algorithm Development and Preliminary Sensitivity Testing

The 2006–2011 interdisciplinary chronic pain rehabilitation patient group consisted of 266 patients. The mean age was 48.0 years and 57.9% were identified as female. After linkages, 256 (97.0%) had at least one physician encounter recording any pain-related diagnostic code in the MCP Claims File and 172 (64.7%) had at least one hospitalization recording at least one pain-related diagnostic code in the NL Discharge Abstract Data (all 16 codes per separation were considered). Twelve people (4.5%) had an entry with the ICD-10-CA code for the diagnosis of acute or chronic pain (R52) in the NL Discharge Abstract Data. After linkages, 96.7% of the 1999–2005 interdisciplinary chronic pain rehabilitation program patient group (*N* = 361, mean age 52.4, 50.1% female), 93.8% of the interdisciplinary chronic pain rehabilitation program waitlist patient group (*N* = 130, mean age 45.6, 64.6% female), and 93.7% of the NL Prescription Drug Plan pain patient group (*N* = 38,532, mean age 61.0, 57.6% female) had at least one encounter or hospitalization recording any pain-related diagnostic code in either the MCP Claims File or the NL Discharge Abstract Data.

Table S5, Supplementary file 2 provides a complete list of possible algorithm combinations considered, the number of each pain patient group identified by each algorithm, and the calculated sensitivities. The algorithm sensitivities were widely variable, ranging from 0.029 to 0.962, depending on the pain patient group and the algorithm restrictiveness. The algorithm sensitivities were lower in the NL Prescription Drug Plan pain patient group than the interdisciplinary chronic pain rehabilitation program patient groups. This is possibly because there is no defined opioid prescription period indicative of long-term use (e.g., 90 days) in the inclusion criteria for the NL Prescription Drug Plan pain patient group.

The first 33 algorithms applied to the administrative data for the pain patient groups explored whether known chronic pain cases could be identified from the administrative data in the full data period (1999–2010) time window via physician encounters or hospital admissions recording any pain-related diagnostic code for up to five unique encounter dates. The next 32 algorithms explored whether (1) known cases of chronic pain could be identified in administrative data while meeting the 6-month temporal criterion in the full data period time window for up to five physician encounter or hospitalization dates recording any pain-related diagnostic code and (2) the inclusion of hospital admission dates recording any pain-related diagnostic code significantly improved identification of known chronic pain cases in the full data period time window. Combining hospital admission dates with fee-for-service physician encounter dates to satisfy the 6-month temporal criterion was a complex process and minimally improved case ascertainment. However, including hospital admission dates recording any pain-related diagnostic code by a medical specialist to satisfy the medical specialist encounter criterion significantly improved case ascertainment in those tested algorithms. In the interest of parsimony, no hospital admission dates recording any pain-related diagnostic codes were included for algorithm validation, except for the algorithms requiring a medical specialist encounter where hospital admission dates with a medical specialist recording any pain-related diagnostic code could satisfy this criterion. The next 56 algorithms explored whether known cases of chronic pain could be identified if the observation window was defined (1- to 7-year observation windows) while meeting the 6-month temporal criterion for a defined number of encounter dates recording any pain-related diagnostic code (two to five dates). The final 56 algorithms explored whether including the MCP physician procedure billing codes reserved for anesthesiologist-delivered intervention treatments in a hospital-based chronic pain clinic would have an impact on the utility of the previous 56 algorithms. The final 56 algorithms had the best performance and were selected for the final validation step.

### Algorithm Validation and Selection

The most performant 56 administrative data chronic pain algorithms from the administrative data algorithm development step were tested against the reference standard in the reference standard cohort. Table S6, Supplementary file 3 provides the tested algorithms and their validation statistics.

The highest sensitivity (0.917; 95% CI, 0.906–0.928) resulted from the least restrictive algorithm requiring the lowest required number of encounter dates recording any pain-related diagnostic code (≥2) in the longest observation time window (7 years). Algorithm sensitivity decreased as the number of required encounter dates increased, the observation time window decreased, or the medical specialist encounter criterion was added. The algorithm with the highest sensitivity had the lowest specificity (0.332; 95% CI, 0.326–0.339) and the highest false positive rate (0.668). The negative predictive value (ranging from 0.783 to 0.925) and the likelihood ratio negative (ranging from 0.852 to 0.249) followed the same trend as the sensitivity.

The highest specificity (0.938; 95% CI, 0.929–0.947) resulted from the most restrictive algorithm requiring the highest number of encounter dates recording any pain-related diagnostic code (≥5) in the shortest observation time window (1 year) and requiring an encounter with a medical specialist recording any pain-related diagnostic code. Algorithm specificity decreased as the number of required encounter dates decreased, the observation time window increased, or the specialist encounter criterion was removed. The algorithm with the highest specificity had the lowest sensitivity (0.200; 95% CI, 0.184–0.216) and the lowest false positive rate (0.062). The positive predictive value (ranging from 0.309 to 0.513) and the likelihood ratio positive (ranging from 1.374 to 3.241) followed the same trend as the specificity. The intersection of sensitivity and specificity plot lines was observed at approximately 0.67 (Figure S1, Supplementary file 4).

The area under the receiver operating characteristic curve ranged from poor (0.569; 95% CI, 0.555–0.583) to acceptable (0.690; 95% CI, 0.678–0.702) selection accuracy of the chronic pain algorithms.^84^ The kappa agreement between the administrative data algorithms and the CPCSSN reference standard ranged from slight (0.150; 95% CI, 0.137–0.163) to fair (0.303; 95% CI, 0.289–0.317).^85^

The most performant algorithm was chosen based on (1) the sensitivity and specificity being closest to 0.67 (the intersection of the sensitivity and specificity plot lines), (2) the best concurrent positive predictive value, and (3) the consensus of the research team regarding the algorithm functionality in assessing the disease burden of chronic pain. Considering the study’s goal and the validation test results, the most performant chronic pain algorithm to identify chronic pain cases in residents attending fee-for-service physician care for pain-related conditions in NL was determined to be (1) a single encounter date with an anesthesiologist recording a chronic pain-related provincial MCP procedure billing code in the MCP Claims File OR (2) five or more physician encounter dates recording any pain-related diagnostic code in a 5-year period with more than 183 days separating at least two pain-related encounter dates in the MCP Claims File. This algorithm identified 42.3% of the reference standard cohort and 37.6% of the 584,875 people in the provincial cohort. Each cohort member selected by the algorithm had a mean of 2.7, a median of 3, and a mode of 3 unique pain-related diagnostic codes recorded in the five required encounter dates. The five most common and five least common ICD-9 pain-related diagnostic codes recorded in the five required encounter dates for algorithm selection are provided in Table S7, Supplementary file 4.

The chronic pain algorithm had 0.703 (95% CI, 0.685–0.722) sensitivity, 0.668 (95% CI, 0.657–0.678) specificity, 0.408 (95% CI, 0.393–0.423) positive predictive value, 0.874 (95% CI, 0.865–0.882) negative predictive value, 2.117 (95% CI, 2.030–2.207) likelihood ratio positive, 0.444 (95% CI, 0.474–0.417) likelihood ratio negative, 4.763 (95% CI, 4.308–5.267) diagnostic odds ratio, 0.685 (95% CI, 0.673–0.698) area under the receiver operating characteristic curve (or adequate indicator of selection accuracy),^84^ and 0.298 (95% CI, 0.285–0.312) kappa agreement (or fair).^85^ The chronic pain algorithm had 0.601–0.868 sensitivity in the pain patient groups.

Of the 2435 false-positive cases, 1794 (73.7%) had at least one encounter with a specialist for any pain-related condition and 34 (1.4%) attended an organized pain clinic for treatment for chronic pain. In addition, 758 (31.1%) false-positive cases were identified by the chronic pain algorithm in administrative data prior to (but not within) the date range of the CPCSSN-NL data. Of the 708 false-negative cases, only 66 (9.3%) did not have at least one encounter in the MCP Claims Data recording any pain-related diagnostic code, and 166 (23.4%) did not meet the benchmark of more than 6 months between at least two encounter dates recording any pain-related diagnostic code. In addition, 651 (62.9%) false-negative cases would be selected if fewer treatments were required and/or the observation time window was longer (i.e., a less restrictive algorithm).

The chronic pain algorithm was tested further for selection accuracy in the age and sex strata of the reference standard cohort (Table 2). In summary, the chronic pain algorithm had lower sensitivity and higher specificity in selecting people aged 34 and younger and higher sensitivity and lower specificity in selecting people aged 65 and over when compared to its selection performance in the overall reference standard cohort.

**Table 2.** Selection accuracy of the chronic pain algorithm^a^ in reference standard cohort age and sex strata

	Prevalence defined by reference standard (%)	Prevalence defined by chronic pain algorithm (%)	Sensitivity	Specificity	PPV	NPV	LR+	LR−	DOR	Kappa
Reference standard cohort	24.6	42.3	0.703	0.668	0.408	0.874	2.12	0.44	4.76	0.30
Age group										
0–14	4.1	7.6	0.250	0.931	0.133	0.967	3.63	0.81	4.51	0.13
15–24	11.7	21.2	0.346	0.805	0.190	0.903	1.77	0.81	2.18	0.11
25–34	16.5	34.2	0.576	0.705	0.279	0.894	1.95	0.60	3.26	0.20
35–44	22.0	38.3	0.635	0.689	0.366	0.870	2.04	0.53	3.86	0.25
45–54	28.7	48.6	0.747	0.619	0.441	0.859	1.96	0.41	4.80	0.30
55–64	35.8	52.2	0.738	0.600	0.507	0.805	1.85	0.44	4.24	0.31
65–79	35.9	59.0	0.780	0.516	0.475	0.807	1.61	0.43	3.79	0.26
80+	31.0	64.8	0.815	0.427	0.390	0.838	1.42	0.43	3.29	0.19
Sex										
Male	20.8	36.6	0.652	0.709	0.371	0.886	2.24	0.49	4.58	0.28
Female	28.1	47.7	0.738	0.625	0.435	0.859	1.97	0.42	4.69	0.30

^a^The most performant chronic pain algorithm was defined as (1) a single encounter date with an anesthesiologist recording a chronic pain–related provincial Medical Care Plan procedure billing code (Table S4, Supplementary file 1) in the Medical Care Plan Fee-for-Service Physicians Claims File OR (2) five or more encounter dates recording any pain-related diagnostic code (Table S3, Supplementary file 1) in a 5-year period with more than 183 days separating at least two pain-related encounter dates in the Medical Care Plan Fee-for-Service Physicians Claims File.

PPV = positive predictive value; NPV = negative predictive value; LR+ = likelihood ratio positive; LR− = likelihood ratio negative; DOR = diagnostic odds ratio.

## Discussion

There is a critical need to determine the societal burden of chronic pain.^1,5,6,19,22^ A validated administrative data algorithm to estimate the epidemiology of chronic pain not only enables financial estimates to be determined^86^ but also enables assessment of the effects of change to health care and population health policy.^78^ To help answer policy-level questions being posed,^32^ this study was undertaken to develop and test an algorithm to identify cases of chronic pain as a single chronic disease using Canadian health administrative data. By linking data from known chronic pain patient groups and a general population group over an 11-year study period, a chronic pain algorithm was created and its selection performance was assessed at 0.703 sensitivity, 0.668 specificity, and 0.408 positive predictive value. Though no tested algorithm met the study goal of ≥0.70 sensitivity, specificity, and positive predictive value, the algorithm deemed best at ascertaining cases of chronic pain from MCP Claims File data to be used for future study was (1) a single encounter date with an anesthesiologist recording a chronic pain–related provincial MCP procedure billing code in the MCP Claims File OR (2) five or more encounter dates with a physician recording any pain-related diagnostic code in a 5-year period with more than 183 days separating at least two pain-related encounter dates in the MCP Claims File. This algorithm satisfied both spatial and temporal benchmarks consistent with the diagnosis of chronic pain.^11,25,37,47,48^ The algorithm selected 37.6% of an NL population cohort from health administrative data.

### Achieving Best Case Ascertainment

The chronic pain algorithm validation performance was comparable to other validation studies assessing health administrative data algorithms for specific chronic pain conditions with respect to the ascertainment measures of sensitivity and specificity. Algorithms identifying cases of neck and back disorders had the best and most consistent performance on tests of selection accuracy (up to 0.71 sensitivity, 0.89 specificity, and 0.83 positive predictive value).^41^ That study’s population included only people with known chronic pain diagnoses, unlike our study. A validation study examining administrative data of survey respondents found very good specificity (>0.90) but poor sensitivity (0.20–0.55) for arthritis case definitions.^78^ Algorithms for other specific and less common chronic pain conditions performed less consistently on validation testing. These included fibromyalgia (0.32–0.42 sensitivity, 0.94–0.97 specificity),^41^ painful neuropathy (0.22–0.39 sensitivity, 0.58–0.80 specificity),^28,41^ chronic regional pain syndrome (0.04–0.07 sensitivity, 0.93–0.98 specificity),^41^ and irritable bowel syndrome (0.112–0.989 sensitivity).^59,87,88^ Choice of codes, frequency criteria, and validation cohort contributed to variability in the validation results of these studies. Because no other study reported validation of administrative data algorithms for chronic pain as a single disease, the present study will form the benchmark against which future studies validating chronic pain algorithms will be compared.

### Ascertainment versus Accuracy

The present study overcame significant challenges to create and validate an administrative data algorithm for chronic pain that included all necessary spatial and temporal benchmarks. Because there is no measurable objective diagnostic test and no consistent agreement among experts on the diagnostic criteria for chronic pain there was a less explicit reference standard against which to compare the chronic pain administrative data algorithms.^1,42^ Algorithm development was further complicated by the discord among physicians regarding best treatment practices for chronic pain conditions,^1,42^ as evidenced by the high number of unique three-digit ICD-9 (67 in total) and ICD-10-CA (83 in total) codes used to identify pain-related conditions in the NL administrative data. The chronic pain algorithm identified a high number of false-positive and false-negative cases, which negatively impacted the selection accuracy tests of positive predictive value, likelihood ratio, and area under the receiver operating characteristic curve. Because the goal of this study was to create an administrative data algorithm to eventually measure the disease burden of chronic pain in the general population, more weight was placed on ascertainment measures (i.e., sensitivity and specificity) than on selection accuracy measures.^43,47,78^ As such, the chronic pain algorithm is better suited for assessment of disease distribution and measuring strength of association with other captured administrative data information in Newfoundland and Labrador than assessment of causation, adverse events, and intervention effectiveness.^47^

### Algorithm Validity to Study Chronic Pain Distribution

The chronic pain algorithm identified 42.3% of the reference standard cohort, which was higher than the 24.6% identified by the reference standard. The high number of false positives identified by the algorithm influenced this discrepancy. When considering the overrepresentation of people 65 years and older in the reference standard cohort, it is possible that the reference standard underascertained cases of chronic pain. Selection accuracy results may also be discordant with clinical reality because nearly 74% of false-positive cases had at least one encounter with a medical specialist for any pain-related condition. This may indicate that many people receiving care for their chronic pain condition from a specialist may no longer have their pain addressed by their primary care physician. The identification of 37.6% in the NL provincial cohort by the chronic pain algorithm was comparable to the 36% chronic pain prevalence in Atlantic Canada (which includes NL) reported by a survey in 2007 but higher than the 21.5% Atlantic Canada prevalence reported in 2011 by another survey.^12,16^ Poor kappa agreement between survey data and administrative data for identifying cases of a pain condition was previously reported and may influence this observation.^87^ Although disagreement between administrative data and medical record or survey data exists, the chronic pain algorithm applied to population-based, widespread administrative data will provide an accurate reflection of geographic and demographic variation of chronic pain distribution in residents attending encounters with fee-for-service physicians in NL.^86^

### Strengths and Limitations

The main strength of this study lies in its methodology that followed established guidelines.^25^ First, the spatial and temporal patterns in the administrative data of patient groups known to have chronic pain were studied to develop the preliminary chronic pain algorithms. The algorithms were then validated by calculating multiple tests of selection accuracy in a general population cohort whose demographics approximated that of the NL general population.^25,54^ Using the CPCSSN electronic medical record data to apply the reference standard provided comprehensive clinical information for a sufficient sample size to test sensitivity and specificity of multiple algorithms with 0.02 precision and 0.05 alpha that was economical in terms of funding and human resources when compared to a manual chart audit. Finally, a broad range of validation statistics obtained from testing a large number of administrative data algorithms using different criteria were reported. These can inform future studies on chronic pain that plan to use health administrative data to achieve different research goals.

There were several limitations to this study. A chronic limitation for all validation studies involving administrative and medical records data is the dependence of its quality on the accuracy of data entry at source.^4,25,58^ Algorithm development and case ascertainment may have been impacted by the noncapture of pain-related treatments delivered by allied health professionals, salaried physicians, or those funded by a third party (such as workers’ compensation) and the allowance of only one diagnostic code entry per episode of care per practitioner (a non-pain-related diagnostic code might have been the chosen code entry for a particular visit even if a pain condition was assessed/treated).^41^ There was differential misclassification bias of the chronic pain algorithm in age groups 34 years and under and 65 years and older, possibly impacting algorithm generalizability in studying chronic pain distribution in these age ranges. Chronic pain prevalence is lower in the younger age groups and higher in the older age groups, which, when combined with the age demographics of the pain patient populations used to develop the preliminary algorithms, factor into the age-related misclassification bias.^3,12–15,25,89^ Though the CPCSSN electronic medical record data were determined to be a valid proxy to manual chart audits for the eight chronic diseases with previously validated CPCSSN case definitions (i.e., hypertension, diabetes mellitus, depression, chronic obstructive pulmonary disease, osteoarthritis, dementia, epilepsy, and Parkinsonism),^57,58^ it was not specifically assessed for chronic pain, which may impact validation results. Finally, the chronic pain algorithm may bias estimates of disease risk to the NL general population (through measures of incidence and prevalence) or disease burden on the NL health system (through measures of association) associated with chronic pain.^25,47^ Any disease risk or burden estimates obtained from using the chronic pain algorithm should be adjusted for this bias as effectively as possible (which may be complex, requiring multiple variables).^47^ If this is not possible, the risk of bias should be explicitly acknowledged and the resultant estimates should be interpreted with caution.

### Generalizability and Future Research

The nature of the NL administrative data and the chronic pain algorithm selection accuracy performance limits its generalizability to extracting disease burden information on residents attending encounters for pain-related conditions with fee-for-service physicians in NL. Validation of the chronic pain algorithm in target population administrative data is recommended prior to its use in non-NL jurisdictions. The required 5-year observation window reduces the practicality of the algorithm for ongoing disease surveillance (due to the long longitudinal data period required to accommodate algorithm application and the recommended 4- to 7-year lead-in period for incidence rate calculations)^43,71^ and reduces the sensitivity of the algorithm to assess the impact of critical societal events (e.g., global pandemic) on chronic pain incidence. The methodology used in this study is generalizable to other Canadian jurisdictions due to similarities in the structure of provincial/territorial physician claims and hospital discharge abstract data.^34^

This is the first study in Canada to derive and validate a health administrative data algorithm for chronic pain as a single chronic disease. To increase algorithm generalizability and maximize the potential of this data source in chronic pain research, future studies are recommended. Future research recommendations include deriving more flexible algorithms to reduce differential misclassification bias based on age, adapting ICD and procedure code lists to specific jurisdictions, assessing the impact of including available administrative pharmacy and allied health data, and exploring the impact of including other medications and procedures used for pain treatment. In the absence of a gold standard objective diagnostic test to confirm the presence of chronic pain, it is recommended that a reference standard with a practical, robust set of criteria be developed and validated for future use in comprehensive health records, electronic medical records, and cleaned electronic medical record data sets (such as CPCSSN).

## Conclusions

The present study sought to derive and validate an algorithm that identifies cases of chronic pain from provincial administrative data in Canada. The chronic pain algorithm aligned with both spatial and temporal frequency benchmarks indicative of a chronic pain diagnosis and was the most performant algorithm based on available data to identify cases of chronic pain from residents attending fee-for-service physician encounters for pain-related conditions in NL. The recommended applications of the chronic pain algorithm include assessment of geographic and demographic variation in disease distribution and assessment of strength of association with other NL administrative data–derived variables (such as health service use and comorbid conditions). Though selection accuracy results preclude use of the chronic pain algorithm for evaluation of interventions, adverse events, and causation, a more restrictive algorithm validated in this study might be considered a more viable option for such research. Further investigation is indicated to fully realize the potential of health administrative data as a valid and efficient source of information to study epidemiology, health care utilization, long-term health outcomes, and effectiveness of policy/health service delivery change associated with chronic pain.

## Supplementary Material

Supplemental Material

## Data Availability

The data that support the findings of this study are securely held at the NL Centre for Health Information and the Eastern Regional Health Authority but restrictions apply to the availability of these data, which were used under data-sharing agreements for the current study and so are not publicly available. Data are, however, available from the NL Centre of Health Information if prespecified criteria are met following its information request procedures. The study author (HF) may be contacted for the data set creation plan. https://www.nlchi.nl.ca/index.php/quality-information/information-requests/record-level-information
