# Supplementary material for: Identifying cases of chronic pain using health administrative data: A validation study
Source: Can J Pain. 2020 Dec 3;4(1):252–67. doi: 10.1080/24740527.2020.1820857 (PMC7967902; doi:10.1080/24740527.2020.1820857)
Supplement: Supplemental Material [file UCJP_A_1820857_SM7355.zip › Supplementary file 1 changes accepted.docx]

**Supplementary materials:** Medication codes, diagnostic codes, and provincial procedure codes used in the Canadian Primary Care Sentinel Surveillance Network-Newfoundland and Labrador data, the Newfoundland and Labrador Prescription Drug Plan data, the Newfoundland and Labrador Medical Care Plan Fee-for-Service Physicians Claims File data, and/or the Newfoundland and Labrador Provincial Discharge Abstract hospital data

**Table S1** Anatomical Therapeutic Classification codes^a^ of opioid medication used almost exclusively for pain treatment

| ATC Code | Drug Name |
| --- | --- |
| N02AA01 | Morphine |
| N02AA02 | Opium |
| N02AA03 | Hydromorphone |
| N02AA05 | Oxycodone |
| N02AA55 | Oxycodone, combinations (Targin®) |
| N02AA59 | Codeine, combinations excluding psycholeptics |
| N02AA79 | Codeine, combinations with psycholeptics |
| N02AB02 | Pethidine (Meperidine in Canada) |
| N02AB03 | Fentanyl |
| N02AC04 | Dextropropoxphene (Discontinued in Canada in 2010) |
| N02AD01 | Pentazocine |
| N02AE01 | Buprenorphine |
| N02AF01 | Butorphanol |
| N02AF02 | Nalbuphine (usually used preoperation or during labour) |
| N02AX02 | Tramadol |
| N02AX06 | Tapentadol (available in Canada since 2010) |
| N02AX52 | Tramadol, combinations |
| N01AH01 | Fentanyl |
| N01AH02 | Alfentanil |
| N01AH03 | Sufentanil |
| N01AH06 | Remifentanil |

Notes: a. WHO ATC/DDD Index; 2012. Available from: <http://www.whocc.no/atc_ddd_index/>. Accessed October 23, 2013.

Abbreviations: ATC; Anatomical Therapeutic Chemical classification codes, WHO; World Health Organization, DDD; defined daily dose.

**Table S2** Pain-related diagnostic codes used in Canadian Primary Care Sentinel Surveillance Network^a^-NL electronic medical record data

| Pain Conditions | ICD-9 codes |
| --- | --- |
|  |  |
| Neuropathic pain | 53, 53.11, 53.9 |
|  | 250.6 |
|  | 256, 256.3, 256.4 |
|  | 350, 350.1, 350.2 |
|  | 351, 351.0, 351.8 |
|  | 352, 352.0, 352.9 |
|  | 353, 353.0, 353.2, 353.6 |
|  | 354, 354.0, 354.4, |
|  | 355, 355.0, 355.1, 355.3, 355.5, 355.6, 355.9, |
|  | 356, 356.2, 356.9, |
|  | 357, 357.2, 357.4, |
|  | 729, 729.0, 729.1, 729.2, 729.3, 729.31, 729.4, 729.5, 729.71, 729.81, 729.82, 729.9 |
|  |  |
| Musculoskeletal conditions and arthritis | 710, 710.0, 710.1, 710.2, 710.4 |
|  | 711.9 |
|  | 712.2, 712.3 |
|  | 713.3, 713,6 |
|  | 714, 714.0, 714.1, 714.3, 714.8, 714.9 |
|  | 715, 715.26, 715.3, 715.9, 715.98 |
|  | 716, 716.1, 716.15, 716.4, 716.5, 716.9 |
|  | 717, 717.0, 717.3, 717.4, 717.41, 717.43, 717.5, 717.6, 717.7, 717.8, 717.82 |
|  | 718, 718.01, 718.07, 718.4, 718.6, 718.80 |
|  | 719, 719.0, 719.1, 719.4, 719.41, 719.44, 719.45, 719.46, 719.47, 719.51, 719.52, 719.53, 719.54, 719.55, 719.56, 719.57, 719.61, 719.62, 719.63, 719.67, 719.7, 719.9 |
|  | 725 |
|  | 726, 726.0, 726.1, 726.11, 726.12, 726.19, 726.3, 726.31, 726.32, 726.33, 726.5, 726.6, 726.61, 726.64, 726.65, 726.7, 726.71, 726.72, 726.79, 726.9, 726.91 |
|  | 727, 727.0, 727.03, 727.05, 727.1, 727.3, 727.4, 727.43, 727.51, 727.6, 727.60, 727.61, 727.62, 727.65, 727.68, 727.82, 727.9 |
|  | 728, 728.10, 728.11, 728.2, 728.3, 728.4, 728.5, 728.6, 728.7, 728.71, 728.79, 728.83, 728.84, 728.85, 728.87, 728.9 |
|  |  |
| Back/neck disorders | 720, 720.0, 720.2, 720.8, 720.9 |
|  | 721, 721.0, 721.2, 721.3, 721.8 |
|  | 722, 722.10, 722.4, 722.5, 722.52, 722.6, 722.71, 722.9, 722.91, 722.92, 722.93 |
|  | 723, 723.0, 723.1, 723.5, 723.8 |
|  | 724, 724.0, 724.02, 724.1, 724.2, 724.3, 724.5, 724.6, 724.7, 724.79, 724.8 |
|  | 737, 737.0, 737.1, 737.12, 737.21, 737.3 |
|  | 738, 738.0, 738.1, 738.5, 738.7 |
|  | 739, 739.5 |
|  | 756, 756.1, 756.11, 756.17, 756.52, 756.59, 756.71 |
|  | 805, 805.6, 805.8 |
|  | 806.0, 806.7 |
|  | 839.8 |
|  | 846, 846.0, 846.1 |
|  | 847, 847.0, 847.2, 847.3, 847.4, 847.9 |
|  | 848, 848.0, 848.1, 848.3, 848.4, 848.41, 848.42, 848.9 |
|  | 905.9 |
|  | 959.09 |
|  |  |
| Bone disorders | 730.0, 730.1, 730.36, 730.38, 730.39 |
|  | 731, 731.0 |
|  | 732, 732.4, 732.7, 732.9 |
|  | 733, 733.0, 733.02, 733.03, 733.2, 733.4, 733.5, 733.6, 733.82, 733.9, 733.90, 733.92, 733.93 |
|  | 734 |
|  | 735, 735.0, 735.2, 735.4, 735.5, 735.8, 735.9 |
|  | 736.00, 736.1, 736.2, 736.21, 736.4, 736.41, 736.73, 736.76, 736.79, 736.81 |
|  |  |
| Musculoskeletal trauma | 807, 807.0, 807.2 |
|  | 808 |
|  | 820 |
|  | 821.00 |
|  | 822 |
|  | 823, 823.02, 823.80, 823.81, 823.82 |
|  | 824, 824.0, 824.2, 824.4, 824.6 |
|  | 825, 825.0, 825.23 |
|  | 826 |
|  | 827 |
|  | 829 |
|  | 831, 831.04 |
|  | 840, 840.0, 840.4, 840.5, 840.6, 840.9 |
|  | 841 |
|  | 842, 842.0, 842.1 |
|  | 843, 843.0 |
|  | 844, 844.0, 844.1, 844.2 |
|  | 845, 845.0, 845.01, 845.03, 845.09, 845.1, 845.11, 845.12, 845.13 |
|  |  |
| Headaches | 346, 346.0, 346.1, 346.2, 346.8, 346.9 |
|  | 784, 784.0, 784.1, 784.9, 784.99 |
|  |  |
| Other conditions associated with chronic pain | 287.2 |
|  | 307, 307.81 |
|  | 337, 337.2 |
|  | 340 |
|  | 349 |
|  | 388.5 |
|  | 440, 440.21, |
|  | 443, 443.0, 443.1, 443.89, 443.9 |
|  | 447.6 |
|  | 459.81, 459.9 |
|  | 524.6 |
|  | 558 |
|  | 564, 564.1, 564.2, 564.4, 564.6, 564.8 |
|  | 569.42 |
|  | 577.0, 577.1 |
|  | 592, 592.0, 592.1 |
|  | 596.59 |
|  | 608.9 |
|  | 617, 617.9 |
|  | 625, 625.0, 625.1, 625.2, 625.3, 625.4, 625.5, 625.6, 625.9 |
|  | 709.2 |
|  | 781, 781.0, 781.1, 781.2, 781.3, 781.94 |
|  | 785.6 |
|  | 786.5, 786.59, 786.8 |
|  | 788.0, 788.1 |
|  | 789, 789.0, 789.06, 789.1, 789.2, 789.3, 789.5, 789.66, 789.9 |
|  | 991.2, 991.3 |
|  |  |
| Central Pain Syndrome, Chronic Pain, or Chronic Pain Syndrome | 338.0^b^, 338.2^b^, 338.4 |

Notes: a. The Canadian Primary Care Sentinel Surveillance Network is a clinical data source comprised of information retrieved directly from the electronic medical records of consenting patients attending participating primary care practices across Canada; b. The diagnostic code is not used in the Canadian Primary Care Sentinel Surveillance Network-Newfoundland and Labrador data but is included for completeness.

Abbreviations: NL, Newfoundland and Labrador; ICD-9, *International Classification of Disease – 9^th^ revision*

**Table S3** Pain-related diagnostic codes used in Newfoundland and Labrador, Canada health administrative data^a^

| ICD-9 codes^b,c^ | Description | ICD-10-CA codes^b^ | Description |
| --- | --- | --- | --- |
|  |  |  |  |
| 53 | Herpes Zoster | F45 | Somatoform Disorders |
| 256 | Ovarian Dysfunction | G43 | Migraine |
| 307 | Special Symptoms or Syndromes, Not Elsewhere Classified | G44 | Other Headache Syndromes |
| 338^d^ | Pain, not elsewhere classified | G50 | Disorders of Trigeminal Nerve |
| 346 | Migraine | G52 | Disorders of Other Cranial Nerves |
| 350 | Trigeminal Nerve Disorders | G53 | Cranial Nerve Disorder in Diseases Classified Elsewhere |
| 351 | Facial Nerve Disorders | G54 | Nerve Root and Plexus Diseases |
| 352 | Disorders of Other Cranial Nerves | G55 | Nerve Root and Plexus Compression in Diseases Classified Elsewhere |
| 353 | Nerve Root and Plexus Disorders | G56 | Mononeuropathies of Upper Limb |
| 354 | Mononeuritis of Upper Limb and Mononeuritis Multiplex | G57 | Mononeuropathies of Lower Limb |
| 355 | Mononeuritis of Lower Limb | G58 | Other Mononeuropathies |
| 356 | Hereditary and Idiopathic Peripheral Neuropathy | G59 | Mononeuropathy in Diseases Classified Elsewhere |
| 357 | Inflammatory and Toxic Neuropathy | G60 | Hereditary and Idiopathic Neuropathy |
| 564 | Functional Digestive Disorders, Not Elsewhere Classified | G61 | Inflammatory Polyneuropathy |
| 625 | Pain and Other Symptoms Associated with Female Genital Organs | G62 | Other and Unspecified Polyneuropathies |
| 710 | Diffuse Diseases of Connective Tissue | G63 | Polyneuropathy in Diseases Classified Elsewhere |
| 711 | Arthropathy Associated with Infections | G64 | Other Diseases of the Peripheral Nervous System |
| 712 | Crystal Arthropathies | G82 | Paraplegia (Paraparesis) and Quadriplegia (Quadriparesis) |
| 713 | Arthropathy Associated with Other Disorders Classified Elsewhere | G89^d^ | Pain, not elsewhere classified |
| 714 | Rheumatoid Arthritis and Other Inflammatory Polyarthropathies | G96 | Other Disorders of Central Nervous System |
| 715 | Osteoarthritis and Allied Disorders | G97 | Intraoperative and Post-procedural Complications and Disorders of Nervous System, Not Elsewhere Classified |
| 716 | Other and Unspecified Arthropathies | L89 | Pressure Ulcer |
| 717 | Internal Derangement of Knee | L97 | Non-Pressure Chronic Ulcer of Lower Limb, Not Elsewhere Classified |
| 718 | Other Derangement of Joint | L98 | Other Disorders of Skin and Subcutaneous Tissue, Not Elsewhere Classified |
| 719 | Other and Unspecified Disorders of Joint | M05 | Rheumatoid Arthritis with Rheumatoid Factor |
| 720 | Ankylosing Spondylitis and Other Inflammatory Spondylopathies | M06 | Other Rheumatoid Arthritis |
| 721 | Spondylosis and Allied Disorders | M07 | Enteropathic Arthropathies |
| 722 | Intervertebral Disc Disorders | M08 | Juvenile Arthritis |
| 723 | Other Disorders of Cervical Region | M10 | Gout |
| 724 | Other and Unspecified Disorders of Back | M11 | Other Crystal Arthropathies |
| 725 | Polymyalgia Rheumatica | M12 | Other and Unspecified Arthropathy |
| 726 | Peripheral Enthesopathies and Allied Syndromes | M13 | Other Arthritis |
| 727 | Other Disorders of Synovium, Tendon, and Bursa | M14 | Arthropathies in Other Diseases Classified Elsewhere |
| 728 | Disorders of Muscle, Ligament, and Fascia | M15 | Polyosteoarthritis |
| 729 | Other Disorders of Soft Tissue | M16 | Osteoarthritis of Hip |
| 730 | Osteomyelitis, Periostitis, and Other Infections Involving Bone | M17 | Osteoarthritis of Knee |
| 731 | Osteitis Deformans and Osteopathies Associated with Other Disorders Classified Elsewhere | M18 | Osteoarthritis of First Carpometacarpal Joint |
| 732 | Osteochondropathies | M19 | Other and Unspecified Osteoarthritis |
| 733 | Other Disorders of Bone and Cartilage | M22 | Disorder of Patella |
| 734 | Flatfoot | M23 | Internal Derangement of Knee |
| 735 | Acquired Deformities of Toe | M24 | Other Specified Joint Derangements |
| 736 | Other Acquired Deformities of Limbs | M25 | Other Joint Disorder, Not Classified Elsewhere |
| 737 | Curvature of Spine | M36 | Systemic Disorders of Connective Tissue in Diseases Classified Elsewhere |
| 738 | Other Acquired Deformity | M43 | Other Deforming Dorsopathies |
| 739 | Nonallopathic Lesions, Not Elsewhere Classified | M45 | Ankylosing Spondylitis |
| 756 | Other Congenital Musculoskeletal Anomalies | M46 | Other Inflammatory Spondylopathies |
| 781 | Symptoms Involving Nervous and Musculoskeletal Systems | M47 | Spondylosis |
| 784 | Symptoms Involving Head and Neck | M48 | Other Spondylopathies |
| 789 | Other Symptoms Involving Abdomen and Pelvis | M49 | Spondylopathies in Diseases Classified Elsewhere |
| 805 | Fracture of Vertebral Column without mention of Spinal Cord Injury | M50 | Cervical Disc Disorders |
| 806 | Fracture of Vertebral Column with Spinal Cord Injury | M51 | Thoracic, Thoracolumbar, and Lumbosacral Intervertebral Disc Disorders |
| 808 | Fracture of Pelvis | M53 | Other and Unspecified Dorsopathies, Not Elsewhere Classified |
| 830 | Dislocation of Jaw | M54 | Dorsalgia |
| 831 | Dislocation of Shoulder | M65 | Synovitis and Tenosynovitis |
| 832 | Dislocation of Elbow | M70 | Soft Tissue Disorders Related to Use, Overuse, and Pressure |
| 839 | Other, Multiple, and Ill-defined Dislocations | M75 | Shoulder Lesions |
| 840 | Sprains and Strains of Shoulder and Upper Arm | M77 | Other Enthesopathies |
| 841 | Sprains and Strains of Elbow and Forearm | M79 | Other and Unspecified Soft Tissue Disorders, Not Elsewhere Classified |
| 842 | Sprains and Strains of Wrist and Hand | M80 | Osteoporosis with Current Pathological Fracture |
| 843 | Sprains and Strains of Hip and Thigh | M81 | Osteoporosis without Current Pathological Fracture |
| 844 | Sprains and Strains of Knee and Leg | M82 | Osteoporosis in Diseases Classified Elsewhere |
| 845 | Sprains and Strains of Ankle and Foot | M89 | Other Disorders of Bone |
| 846 | Sprains and strains of Sacroiliac Region | M99 | Biomechanical Lesions, Not Elsewhere Classified |
| 847 | Sprains and Strains of Other Unspecified Parts of Back | R07 | Pain in Throat and Chest |
| 848 | Other and Ill-defined Sprains and Strains | R10 | Abdominal and Pelvic Pain |
| 905 | Late Effects of Musculoskeletal and Connective Tissue Injuries | R26 | Abnormalities of Gait and Mobility |
| 907 | Late Effects of Injuries to the Nervous System | R29 | Other Symptoms and Signs Involving the Nervous and Musculoskeletal Systems |
| 908 | Late Effects of Other and Unspecified Injuries | R51 | Headache |
|  |  | R52 | Pain, Unspecified |
|  |  | S12 | Fracture of Cervical Vertebra and Other Parts of Neck |
|  |  | S13 | Dislocation and Sprain of Joints and Ligaments at Neck Level |
|  |  | S22 | Fracture of Rib(s), Sternum, and Thoracic Spine |
|  |  | S32 | Fracture of the Lumbar Spine and Pelvis |
|  |  | S42 | Fracture of the Shoulder and Upper Arm |
|  |  | S43 | Dislocation and Sprain of Joints and Ligaments of Shoulder Girdle |
|  |  | S53 | Dislocation and Sprain of Joints and Ligaments of Elbow |
|  |  | T02 | Fractures Involving Several Regions of the Body |
|  |  | T08 | Fractures of the Spine, Level Not Specified |
|  |  | T85 | Complications of Other Internal Prosthetic Devices, Implants, and Grafts |
|  |  | T88 | Other Complications of Surgical and Medical Care, Not Elsewhere Classified |
|  |  | T91 | Sequelae of Injuries of Neck and Body |
|  |  | T92 | Sequelae of Injuries of Upper Limb |
|  |  | T93 | Sequelae of Injuries of Lower Limb |
|  |  | T94 | Sequelae of Injuries Involving Multiple and Unspecified Body Region |

Notes: a. Newfoundland and Labrador (NL) health administrative datasets used in the study: 1) the Provincial Discharge Abstract Database (NL Discharge Abstract Data), which is the NL component of the Canadian Institute of Health Information national Discharge Abstracts Database, containing information on all separations from acute health care facilities in NL, including admission date and up to 16 diagnostic codes, and 2) Medical Care Plan (MCP) Fee-for-Service Physicians Claims File (MCP Claims File) containing information, including one diagnostic code and one provincial billing code, on all claims for health services provided by fee-for-service physicians in NL; b. NL Discharge Abstract Data used five-digit ICD-9 codes up to March 31, 2001, and six-digit ICD-10(Canadian) codes from April 1, 2001 onwards; c. MCP Claims File data used three-digit ICD-9 codes throughout the data study period; d. The diagnostic code is not used in the NL Discharge Abstract Data or the MCP Claims File data but is included for completeness.

Abbreviations: ICD-9, *International Classification of Disease-9^th^ Revision*; ICD-10-CA, *International Classification of Disease-10^th^ Revision (Canadian)*; NL, Newfoundland and Labrador; MCP, Medical Care Plan

**Table S4** Newfoundland and Labrador, Canada, provincial Medical Care Plan chronic pain clinic procedure billing codes

| Fee Code | Procedure |
| --- | --- |
|  |  |
| 400020 | Pain Clinic Consultation |
| 419020 | Pain Clinic Reassessment |
| 578000 | Epidural Steroid Injection |
| 578020 | Intercostal Nerve Block(s) |
| 578040 | Paravertebral Nerve Block of Thoracic or Lumbar Roots |
| 578060 | Peripheral Nerve Block for Chronic Pain |
| 578080 | Cranial Nerve/Branch Block for Chronic Pain |
| 578100 | Stellate Ganglion Block |
| 578120 | Intravenous Sympathetic Block by Injection and Infusion of Bretylium, Guanetidine, and Reserpine |
| 578140 | Intravenous Injection and Infusion with Lidocaine for the treatment of Chronic Pain |

Source: Medical Care Plan, Department of Health and Community Services. Medical Payment Schedule - 2009. St. John’s (NL): Government of Newfoundland and Labrador; 2011. [accessed 10 Jan 2017]. <http://www.health.gov.nl.ca/health/mcp/providers/full_mcp_payment_schedule_2009.pdf>.
