# Supplementary material for: Identifying cases of chronic pain using health administrative data: A validation study
Source: Can J Pain. 2020 Dec 3;4(1):252–67. doi: 10.1080/24740527.2020.1820857 (PMC7967902; doi:10.1080/24740527.2020.1820857)
Supplement: Supplemental Material [file UCJP_A_1820857_SM7355.zip › Supplementary file 2 changes accepted.docx]

**Table S5** Preliminary chronic pain administrative data algorithms and sensitivity testing in pain patient populations^a^

| Algorithm | 06-11 CPDM Patients | 99-05 CPDM Patients | CPDM Waitlist Patients | NLPDP Pain Patients |
| --- | --- | --- | --- | --- |
|  | N=266 | N=361 | N=130 | N=38532 |
|  | n(sensitivity) | n(sensitivity) | n(sensitivity) | n(sensitivity) |
|  |  |  |  |  |
| 1 PC ever | 95(0.357) | 79(0.219) | 21(0.162) | 1138(0.029) |
| 2 P ever | 255 (0.959) | 340(0.942) | 118(0.908) | 33713(0.875) |
| 2 H ever | 133(0.500) | 149(0.413) | 51(0.392) | 8056(0.209) |
| 2 P OR 2 H ever | 256(0.962) | 340(0.942) | 119(0.915) | 34274(0.889) |
| 2 P OR H ever | 256(0.962) | 342(0.947) | 120(0.923) | 34434(0.894) |
| 3 P ever | 254(0.955) | 336(0.931)) | 114(0.877) | 31941((0.829) |
| 3 H ever | 103(0.387) | 103(0.285) | 26(0.20) | 4672(0.121) |
| 3 P OR 3 H ever | 255(0.959) | 339(0.939) | 114(0.877) | 32619(0.847) |
| 3 P OR H ever | 255(0.959) | 339(0.939) | 117(0.900) | 32929(0.855) |
| 4 P ever | 253(0.951) | 329(0.911) | 112(0.862) | 30310(0.787) |
| 4 H ever | 80(0.301) | 80(0.222) | 21(0.162) | 2961(0.077) |
| 4 P OR 4 H ever | 254(0.955) | 332(0.920) | 112(0.862) | 31193(0.810) |
| 4 P OR H ever | 254(0.955) | 333(0.922) | 113(0.869) | 31559(0.819) |
| 5 P ever | 248(0.932) | 319(0.884) | 109(0.838) | 28738(0.771) |
| 5 H ever | 60(0.226) | 60(0.166) | 15(0.115) | 2003(0.052) |
| 5 P OR 5 H ever | 249(0.936) | 323(0.895) | 109(0.838) | 29786(0.773) |
| 5 P OR H ever | 250(0.940) | 328(0.909) | 109(0.838) | 30190(0.784) |
| 2 P ever AND >/=1 S ever | 244(0.917) | 307(0.850) | 100(0.769) | 24930(0.647) |
| 2 H ever AND >/= 1 S ever | 133(0.500) | 147(0.407) | 51(0.392) | 7868(0.204) |
| 2 P OR 2 H ever AND >/= 1 S ever | 245(0.921) | 307(0.850) | 101(0.777) | 25336(0.658) |
| 2 P OR H ever with >/= 1 S ever | 245(0.921) | 309(0.856) | 101(0.777) | 25465(0.661) |
| 3 P ever AND >/= 1 S ever | 244(0.917) | 305(0.845) | 98(0.754) | 24167(0637) |
| 3 H ever AND >/= 1 S ever | 103(0.387) | 102(0.283) | 26(0.20) | 4598(0.119) |
| 3 P OR 3 H ever AND >/= 1 S ever | 245(0.921) | 307(0.850) | 98(0.754) | 24696(0.641) |
| 3 P OR H ever AND >/= 1 S ever | 245(0.921) | 307(0.850) | 101(0.777) | 24963(0.648) |
| 4 P ever AND >/= 1 S ever | 244(0.917) | 302(0.837) | 97(0.746) | 23399(0.607) |
| 4 H ever AND >/= 1 S ever | 80(0.301) | 79(0.219) | 21(0.162) | 2926(0.076) |
| 4 P OR 4 H ever AND >/= 1 S ever | 245(0.921) | 304(0.842) | 97(0.746) | 24071(0.625) |
| 4 P OR H ever AND >/= 1 S ever | 245(0.921) | 305(0.845) | 98(0.754) | 24392(0.633) |
| 5 P ever AND >/= 1 S ever | 240(0.902) | 295(0.817) | 97(0.746) | 22559(0.585) |
| 5 H ever AND >/= 1 S ever | 60(0.226) | 59(0.163) | 15(0.115) | 1987(0.052) |
| 5 P OR 5 H ever AND >/= 1 S ever | 241(0.906) | 299(0.828) | 97(0.746) | 23403(0.607) |
| 5 P OR H ever AND >/= 1 S ever | 242(0.910) | 304(0.842) | 97(0.746) | 23761(0.617) |
| 2 P ever AND > 183 days between 2 P | 253(0.951) | 338(0.936) | 117(0.900) | 32829(0.852) |
| 2 H ever AND P > 183 days between 2 H | 133(0.500) | 149(0.413) | 51(0.392) | 7959(0.207) |
| 2 P OR 2 H ever AND > 183 days between 2 P or 2 H | 245(0.921) | 338(0.936) | 118(0.908) | 33085(0.859) |
| 2 P OR H ever AND > 183 days between 2 P or H | 254(0.955) | 340(0.942) | 119(0.915) | 33220(0.862) |
| 3 P ever AND > 183 days between 2 P | 253(0.951) | 335(0.928) | 113(0.869) | 31586(0.820) |
| 3 H ever AND > 183 days between 2 H | 103(0.387) | 103(0.285) | 26(0.200) | 4642(0.120) |
| 3 P OR 3 H ever AND > 183 days between 2 P or 2 H | 254(0.955) | 337(0.934) | 113(0.869) | 32027(0.831) |
| 3 P OR H ever AND P > 183 days between 2 P or H | 254(0.955) | 337(0.934) | 116(0.892) | 32309(0.838) |
| 4 P ever AND > 183 days between 2 P | 252(0.947) | 329(0.911) | 111(0.854) | 30134(0.782) |
| 4 H ever AND > 183 days between 2 H | 80(0.301) | 80(0.222) | 21(0.162) | 2952(0.077) |
| 4 P OR 4 H ever AND P > 183 days between 2 P or 2 H | 253(0.951) | 331(0.917) | 111(0.854) | 30831(0.800) |
| 4 P OR H ever AND > 183 days between 2 P or H | 253(0.951) | 332(0.920) | 112(0.862) | 31179(0.809) |
| 5 P ever AND > 183 days between 2 P | 247(0.929) | 319(0.884) | 108(0.831) | 28645(0.743) |
| 5 H ever AND > 183 days between 2 H | 60(0.226) | 60(0.166) | 15(0.115) | 2002(0.052) |
| 5 P OR 5 H ever AND > 183 days between 2 P or 2 H | 248(0.932) | 322(0.892) | 108(0.831) | 29542(0.767) |
| 5 P OR H ever AND > 183 days between 2 P or H | 249(0.936) | 327(0.906) | 108(0.831) | 29931(0.777) |
| 2 P ever AND > 183 days between 2 P AND >/= 1 S ever | 244(0.917) | 305(0.845) | 100(0.769) | 24534(0.637) |
| 2 H ever AND > 183 days between 2 H AND >/= 1 S ever | 133(0.500) | 147(0.407) | 51(0.392) | 7791(0.202) |
| 2 P OR 2 H ever AND > 183 days between 2 P or 2 H AND >/= 1 S ever | 245(0.921) | 305(0.845) | 101(0.777) | 24745(0.642) |
| 2 P OR H ever AND > 183 days between 2 P or H AND >/= 1 S ever | 245(0.921) | 307(0.850) | 101(0.777) | 24857(0.645) |
| 3 P AND > 183 days between 2 P AND >/= 1 S ever | 244(0.917) | 304(0.842) | 98(0.754) | 23980(0.622) |
| 3 H ever AND > 183 days between 2 H AND >/= 1 S ever | 103(0.387) | 102(0.281) | 26(0.200) | 4574(0.119) |
| 3 P OR 3 H ever AND > 183 days between 2 P or 2 H AND >/= 1 S ever | 245(0.921) | 305(0.845) | 98(0.754) | 24315(0.631) |
| 3 P OR H ever AND > 183 days between 2 P or H AND >/= 1 S ever | 245(0.921) | 305(0.845) | 101(0.777) | 24555(0.637) |
| 4 P ever AND > 183 days between 2 P AND >/= 1 S ever | 244(0.917) | 302(0.837) | 97(0.746) | 23296(0.605) |
| 4 H ever AND > 183 days between 2 H AND >/= 1 S ever | 80(0.301) | 79(0.219) | 21(0.162) | 2917(0.076) |
| 4 P OR 4 H ever AND > 183 days between 2 P or 2 H AND >/= 1 S ever | 245(0.921) | 303(0.839) | 97(0.746) | 23810(0.618) |
| 4 P OR H ever AND > 183 days between 2 P or H AND >/= 1 S ever | 245(0.921) | 304(0.842) | 98(0.754) | 24116(0.626) |
| 5 P ever AND > 183 days between 2 P AND >/= 1 S ever | 240(0.902) | 295(0.817) | 97(0.746) | 22505(0.584) |
| 5 H ever AND > 183 days between 2 H AND >/= 1 S ever | 60(0.226) | 59(0.163) | 15(0.115) | 1986(0.052) |
| 5 P OR 5 H ever AND > 183 days between 2 P or 2 H AND >/= 1 S ever | 241(0.906) | 298(0.825) | 97(0.746) | 23217(0.603) |
| 5 P OR H ever AND > 183 days between 2 P or H AND >/= 1 S ever | 242(0.910) | 303(0.839) | 97(0.746) | 23562(0.611) |
| 2 P AND > 183 days between 2 P in 1 YR | 148(0.556) | 215(0.596) | 68(0.523) | 15509(0.402) |
| 2 P AND > 183 days between 2 P in 2 YR | 210(0.789) | 289(0.801) | 100(0.769) | 23890(0.620) |
| 2 P AND > 183 days between 2 P in 3 YR | 222(0.835) | 305(0.845) | 108(0.831) | 27213(0.706) |
| 2 P AND > 183 days between 2 P in 4 YR | 237(0.891) | 322(0.892) | 111(0.854) | 29163(0.757) |
| 2 P AND > 183 days between 2 P in 5 YR | 243(0.914) | 328(0.909) | 114(0.877) | 30360(0.788) |
| 2 P AND > 183 days between 2 P in 6 YR | 248(0.932) | 332(0.920) | 116(0.892) | 31185(0.809) |
| 2 P AND > 183 days between 2 P in 7 YR | 249(0.936) | 335(0.928) | 117(0.900) | 31785(0.825) |
| 3 P AND > 183 days between 2 P in 1 YR | 132(0.496) | 192(0.532) | 62(0.477) | 12807(0.332) |
| 3 P AND > 183 days between 2 P in 2 YR | 192(0.722) | 267(0.740) | 86(0.662) | 20617(0.535) |
| 3 P AND > 183 days between 2 P in 3 YR | 213(0.801) | 294(0.814) | 95(0.731) | 24278(0.630) |
| 3 P AND > 183 days between 2 P in 4 YR | 226(0.850) | 314(0.870) | 101(0.777) | 26638(0.691) |
| 3 P AND > 183 days between 2 P in 5 YR | 236(0.887) | 320(0.886) | 107(0.823) | 28178(0.731) |
| 3 P AND > 183 days between 2 P in 6 YR | 248(0.932) | 326(0.903) | 111(0.854) | 29273(0.760) |
| 3 P AND > 183 days between 2 P in 7 YR | 249(0.936) | 329(0.911) | 112(0.862) | 30148(0.782) |
| 4 P AND > 183 days between 2 P in 1 YR | 103(0.387) | 167(0.463) | 51(0.392) | 10043(0.261) |
| 4 P AND > 183 days between 2 P in 2 YR | 171(0.643) | 249(0.690) | 77(0.592) | 17108(0.444) |
| 4 P AND > 183 days between 2 P in 3 YR | 197(0.741) | 274(0.759) | 89(0.685) | 20951(0.544) |
| 4 P AND > 183 days between 2 P in 4 YR | 214(0.805) | 295(0.817) | 94(0.723) | 23746(0.616) |
| 4 P AND > 183 days between 2 P in 5 YR | 229(0.861) | 308(0.853) | 101(0.777) | 25646(0.666) |
| 4 P AND > 183 days between 2 P in 6 YR | 237(0.891) | 316(0.875) | 108(0.831) | 27080(0.703) |
| 4 P AND > 183 days between 2 P in 7 YR | 243(0.914) | 319(0.884) | 109(0.838) | 28152(0.731) |
| 5 P AND > 183 days between 2 P in 1 YR | 78(0.293) | 145(0.402) | 42(0.323) | 7667(0.199) |
| 5 P AND > 183 days between 2 P in 2 YR | 144(0.541) | 223(0.618) | 68(0.523) | 14088(0.366) |
| 5 P AND > 183 days between 2 P in 3 YR | 183(0.688) | 255(0.706) | 81(0.623) | 17961(0.466) |
| 5 P AND > 183 days between 2 P in 4 YR | 202(0.759) | 274(0.759) | 88(0.677) | 20911(0.543) |
| 5 P AND > 183 days between 2 P in 5 YR | 222(0.835) | 293(0.812) | 92(0.708) | 23061(0.598) |
| 5 P AND > 183 days between 2 P in 6 YR | 231(0.868) | 304(0.842) | 100(0.769) | 24795(0.643) |
| 5 P AND > 183 days between 2 P in 7 YR | 238(0.895) | 310(0.859) | 104(0.800) | 26102(0.677) |
| 2 P AND > 183 days between 2 P in 1 YR AND >/= 1 S ever | 144(0.541) | 201(0.557) | 61(0.469) | 12537(0.325) |
| 2 P AND > 183 days between 2 P in 2 YR AND >/= 1 S ever | 204(0.767) | 268(0.742) | 87(0.669) | 18662(0.484) |
| 2 P AND > 183 days between 2 P in 3 YR AND >/= 1 S ever | 216(0.812) | 281(0.778) | 94(0.723) | 20927(0.543) |
| 2 P AND > 183 days between 2 P in 4 YR AND >/= 1 S ever | 231(0.868) | 294(0.814) | 95(0.731) | 22215(0.577) |
| 2 P AND > 183 days between 2 P in 5 YR AND >/= 1 S ever | 236(0.887) | 298(0.825) | 97(0.746) | 22958(0.596) |
| 2 P AND > 183 days between 2 P in 6 YR AND >/= 1 S ever | 240(0.902) | 301(0.834) | 99(0.762) | 23480(0.609) |
| 2 P AND > 183 days between 2 P in 7 YR AND >/= 1 S ever | 241(0.906) | 303(0.839) | 100(0.769) | 23858(0.619) |
| 3 P AND > 183 days between 2 P in 1 YR AND >/= 1 S ever | 128(0.481) | 183(0.507) | 59(0.454) | 10678(0.277) |
| 3 P AND > 183 days between 2 P in 2 YR AND >/= 1 S ever | 187(0.703) | 251(0.695) | 79(0.608) | 16608(0.431) |
| 3 P AND > 183 days between 2 P in 3 YR AND >/= 1 S ever | 208(0.782) | 274(0.759) | 85(0.654) | 19232(0.499) |
| 3 P AND > 183 days between 2 P in 4 YR AND >/= 1 S ever | 221(0.831) | 289(0.801) | 89(0.685) | 20826(0.540) |
| 3 P AND > 183 days between 2 P in 5 YR AND >/= 1 S ever | 230(0.865) | 294(0.814) | 92(0.708) | 21804(0.566) |
| 3 P AND > 183 days between 2 P in 6 YR AND >/= 1 S ever | 240(0.902) | 299(0.828) | 96(0.738) | 22501(0.584) |
| 3 P AND > 183 days between 2 P in 7 YR AND >/= 1 S ever | 241(0.906) | 299(0.828) | 97(0.746) | 23071(0.599) |
| 4 P AND > 183 days between 2 P in 1 YR AND >/= 1 S ever | 102(0.383) | 160(0.443) | 49(0.377) | 8564(0.222) |
| 4 P AND > 183 days between 2 P in 2 YR AND >/= 1 S ever | 168(0.632) | 237(0.657) | 72(0.554) | 14210(0.369) |
| 4 P AND > 183 days between 2 P in 3 YR AND >/= 1 S ever | 194(0.729) | 261(0.723) | 81(0.623) | 17088(0.443) |
| 4 P AND > 183 days between 2 P in 4 YR AND >/= 1 S ever | 210(0.789) | 277(0.767) | 83(0.638) | 19079(0.495) |
| 4 P AND > 183 days between 2 P in 5 YR AND >/= 1 S ever | 225(0.846) | 287(0.795) | 87(0.669) | 20349(0.528) |
| 4 P AND > 183 days between 2 P in 6 YR AND >/= 1 S ever | 232(0.872) | 292(0.809) | 94(0.723) | 21296(0.553) |
| 4 P AND > 183 days between 2 P in 7 YR AND >/= 1 S ever | 237(0.891) | 294(0.814) | 95(0.731) | 22017(0.571) |
| 5 P AND > 183 days between 2 P in 1 YR AND >/= 1 S ever | 78(0.293) | 138(0.382) | 40(0.308) | 6662(0.179) |
| 5 P AND > 183 days between 2 P in 2 YR AND >/= 1 S ever | 141(0.530) | 213(0.590) | 66(0.508) | 11961(0.310) |
| 5 P AND > 183 days between 2 P in 3 YR AND >/= 1 S ever | 180(0.677) | 243(0.673) | 77(0.592) | 15001(0.389) |
| 5 P AND > 183 days between 2 P in 4 YR AND >/= 1 S ever | 199(0.748) | 260(0.720) | 81(0.623) | 17220(0.447) |
| 5 P AND > 183 days between 2 P in 5 YR AND >/= 1 S ever | 218(0.820) | 276(0.765) | 82(0.631) | 18756(0.487) |
| 5 P AND > 183 days between 2 P in 6 YR AND >/= 1 S ever | 227(0.853) | 285(0.789) | 89(0.685) | 19954(0.518) |
| 5 P AND > 183 days between 2 P in 7 YR AND >/= 1 S ever | 233(0.876) | 288(0.798) | 93(0.715) | 20850(0.541) |
| (1 PC) OR (2 P ever AND > 183 days between 2 P AND >/= 1 S ever) | 244(0.917) | 305(0.845) | 100(0.769) | 24388(0.633) |
| (1 PC) OR (3 P ever AND > 183 days between 2 P AND >/= 1 S ever) | 244(0.917) | 304(0.842) | 98(0.754) | 23920(0.621) |
| (1 PC) OR (4 P ever AND > 183 days between 2 P AND >/= 1 S ever) | 244(0.917) | 303(0.839) | 97(0.746) | 23274(0.604) |
| (1 PC) OR (5 P ever AND > 183 days between 2 P AND >/= 1 S ever) | 241(0.906) | 298(0.825) | 97(0.746) | 22501(0.584) |
| (1 PC) OR (2 P AND > 183 days between 2 P in 1 YR) | 191(0.718) | 237(0.657) | 74(0.569) | 15882(0.412) |
| (1 PC) OR (2 P AND > 183 days between 2 P in 2 YR) | 227(0.853) | 295(0.817) | 101(0.777) | 24022(0.623) |
| (1 PC) OR (2 P AND > 183 days between 2 P in 3 YR) | 233(0.876) | 307(0.850) | 108(0.831) | 27275(0.708) |
| (1 PC) OR (2 P AND > 183 days between 2 P in 4 YR) | 240(0.902) | 323(0.895) | 111(0.854) | 29197(0.758) |
| (1 PC) OR (2 P AND > 183 days between 2 P in 5 YR) | 245(0.921) | 328(0.909) | 114(0.877) | 30382(0.788) |
| (1 PC) OR (2 P AND > 183 days between 2 P in 6 YR) | 249(0.936) | 332(0.920) | 116(0.892) | 31200(0.810) |
| (1 PC) OR (2 P AND > 183 days between 2 P in 7 YR) | 250(0.940) | 335(0.928) | 117(0.900) | 31797(0.825) |
| (1 PC) OR (3 P AND > 183 days between 2 P in 1 YR) | 180(0.677) | 217(0.601) | 70(0.538) | 13239(0.344) |
| (1 PC) OR (3 P AND > 183 days between 2 P in 2 YR) | 218(0.820) | 276(0.765) | 89(0.685) | 20800(0.540) |
| (1 PC) OR (3 P AND > 183 days between 2 P in 3 YR) | 230(0.865) | 299(0.828) | 96(0.738) | 24385(0.633) |
| (1 PC) OR (3 P AND > 183 days between 2 P in 4 YR) | 237(0.891) | 316(0.875) | 102(0.785) | 26693(0.693) |
| (1 PC) OR (3 P AND > 183 days between 2 P in 5 YR) | 242(0.910) | 320(0.886) | 107(0.823) | 28211(0.732) |
| (1 PC) OR (3 P AND > 183 days between 2 P in 6 YR) | 249(0.936) | 326(0.903) | 111(0.854) | 29295(0.760) |
| (1 PC) OR (3 P AND > 183 days between 2 P in 7 YR) | 250(0.940) | 329(0.911) | 112(0.862) | 30166(0.783) |
| (1 PC) OR (4 P AND > 183 days between 2 P in 1 YR) | 158(0.594) | 197(0.546) | 59(0.454) | 10559(0.274) |
| (1 PC) OR (4 P AND > 183 days between 2 P in 2 YR) | 205(0.771) | 262(0.726) | 80(0.615) | 17368(0.451) |
| (1 PC) OR (4 P AND > 183 days between 2 P in 3 YR) | 219(0.823) | 281(0.778) | 90(0.692) | 21111(0.548) |
| (1 PC) OR (4 P AND > 183 days between 2 P in 4 YR) | 230(0.865) | 299(0.828) | 95(0.731) | 23843(0.619) |
| (1 PC) OR (4 P AND > 183 days between 2 P in 5 YR) | 237(0.891) | 310(0.859) | 101(0.777) | 25702(0.667) |
| (1 PC) OR (4 P AND > 183 days between 2 P in 6 YR) | 241(0.906) | 317(0.878) | 108(0.831) | 27113(0.704) |
| (1 PC) OR (4 P AND > 183 days between 2 P in 7 YR) | 245(0.921) | 320(0.886) | 109(0.838) | 28174(0.731) |
| (1 PC) OR (5 P AND > 183 days between 2 P in 1 YR) | 143(0.538) | 178(0.493) | 52(0.400) | 8269(0.215) |
| (1 PC) OR (5 P AND > 183 days between 2 P in 2 YR) | 187(0.703) | 238(0.659) | 73(0.562) | 14426(0.374) |
| (1 PC) OR (5 P AND > 183 days between 2 P in 3 YR) | 207(0.778) | 264(0.731) | 82(0.631) | 18173(0.472) |
| (1 PC) OR (5 P AND > 183 days between 2 P in 4 YR) | 220(0.827) | 280(0.776) | 89(0.685) | 21045(0.546) |
| *(1 PC) OR (5 P AND > 183 days between 2 P in 5 YR)^b^* | ***231(0.868)*** | ***297(0.823)*** | ***93(0.715)*** | ***23151(0.601)*** |
| (1 PC) OR (5 P AND > 183 days between 2 P in 6 YR) | 236(0.887) | 307(0.850) | 100(0.769) | 24844(0.645) |
| (1 PC) OR (5 P AND > 183 days between 2 P in 7 YR) | 241(0.906) | 313(0.867) | 104(0.800) | 26135(0.678) |
| (1 PC) OR ((2 P AND > 183 days between 2 P in 1 YR) AND >/= 1 S ever) | 187(0.703) | 223(0.618) | 67(0.515) | 12911(0.335) |
| (1 PC) OR ((2 P AND > 183 days between 2 P in 2 YR) AND >/= 1 S ever) | 221(0.831) | 274(0.759) | 88(0.677) | 18795(0.488) |
| (1 PC) OR ((2 P AND > 183 days between 2 P in 3 YR) AND >/= 1 S ever) | 227(0.853) | 283(0.784) | 94(0.723) | 20990(0.545) |
| (1 PC) OR ((2 P AND > 183 days between 2 P in 4 YR) AND >/= 1 S ever) | 234(0.880) | 295(0.817) | 95(0.731) | 22250(0.577) |
| (1 PC) OR ((2 P AND > 183 days between 2 P in 5 YR) AND >/= 1 S ever) | 238(0.895) | 298(0.825) | 97(0.746) | 22981(0.596) |
| (1 PC) OR ((2 P AND > 183 days between 2 P in 6 YR) AND >/= 1 S ever) | 241(0.906) | 301(0.834) | 99(0.762) | 23496(0.610) |
| (1 PC) OR ((2 P AND > 183 days between 2 P in 7 YR) AND >/= 1 S ever) | 242(0.910) | 303(0.839) | 100(0.769) | 23871(0.620) |
| (1 PC) OR ((3 P AND > 183 days between 2 P in 1 YR) AND >/= 1 S ever) | 176(0.662) | 208(0.576) | 67(0.515) | 11111(0.288) |
| (1 PC) OR ((3 P AND > 183 days between 2 P in 2 YR) AND >/= 1 S ever) | 213(0.801) | 260(0.720) | 82(0.631) | 16797(0.436) |
| (1 PC) OR ((3 P AND > 183 days between 2 P in 3 YR) AND >/= 1 S ever) | 225(0.846) | 279(0.773) | 86(0.662) | 19340(0.502) |
| (1 PC) OR ((3 P AND > 183 days between 2 P in 4 YR) AND >/= 1 S ever) | 232(0.872) | 291(0.806) | 90(0.692) | 20882(0.542) |
| (1 PC) OR ((3 P AND > 183 days between 2 P in 5 YR) AND >/= 1 S ever) | 236(0.887) | 294(0.814) | 92(0.708) | 21838(0.567) |
| (1 PC) OR ((3 P AND > 183 days between 2 P in 6 YR) AND >/= 1 S ever) | 241(0.906) | 299(0.828) | 96(0.738) | 22524(0.585) |
| (1 PC) OR ((3 P AND > 183 days between 2 P in 7 YR) AND >/= 1 S ever) | 242(0.910) | 299(0.828) | 97(0.746) | 23090(0.599) |
| (1 PC) OR ((4 P AND > 183 days between 2 P in 1 YR) AND >/= 1 S ever) | 157(0.590) | 190(0.526) | 57(0.438) | 9081(0.236) |
| (1 PC) OR ((4 P AND > 183 days between 2 P in 2 YR) AND >/= 1 S ever) | 202(0.759) | 250(0.693) | 75(0.577) | 14471(0.376) |
| (1 PC) OR ((4 P AND > 183 days between 2 P in 3 YR) AND >/= 1 S ever) | 216(0.812) | 268(0.742) | 82(0.631) | 17249(0.448) |
| (1 PC) OR ((4 P AND > 183 days between 2 P in 4 YR) AND >/= 1 S ever) | 226(0.850) | 281(0.778) | 84(0.646) | 19177(0.498) |
| (1 PC) OR ((4 P AND > 183 days between 2 P in 5 YR) AND >/= 1 S ever) | 233(0.876) | 289(0.801) | 87(0.669) | 20406(0.530) |
| (1 PC) OR ((4 P AND > 183 days between 2 P in 6 YR) AND >/= 1 S ever) | 236(0.887) | 293(0.812) | 94(0.723) | 21330(0.554) |
| (1 PC) OR ((4 P AND > 183 days between 2 P in 7 YR) AND >/= 1 S ever) | 239(0.898) | 295(0.818) | 95(0.731) | 22040(0.572) |
| (1 PC) OR ((5 P AND > 183 days between 2 P in 1 YR) AND >/= 1 S ever) | 143(0.538) | 171(0.474) | 50(0.385) | 7265(0.189) |
| (1 PC) OR ((5 P AND > 183 days between 2 P in 2 YR) AND >/= 1 S ever) | 184(0.692) | 228(0.632) | 71(0.546) | 12300(0.319) |
| (1 PC) OR ((5 P AND > 183 days between 2 P in 3 YR) AND >/= 1 S ever) | 204(0.767) | 252(0.698) | 78(0.600) | 15214(0.395) |
| (1 PC) OR ((5 P AND > 183 days between 2 P in 4 YR) AND >/= 1 S ever) | 217(0.816) | 266(0.737) | 82(0.631) | 17355(0.450) |
| (1 PC) OR ((5 P AND > 183 days between 2 P in 5 YR) AND >/= 1 S ever) | 227(0.853) | 280(0.776) | 83(0.638) | 18847(0.489) |
| (1 PC) OR ((5 P AND > 183 days between 2 P in 6 YR) AND >/= 1 S ever) | 232(0.872) | 288(0.798) | 89(0.685) | 20004(0.519) |
| (1 PC) OR ((5 P AND > 183 days between 2 P in 7 YR) AND >/= 1 S ever) | 236(0.887) | 291(0.806) | 93(0.715) | 20884(0.542) |

Notes: a. Inclusion criteria for the pain populations were: 1) attending an interdisciplinary chronic pain rehabilitation program from 2006-2011, 2) attending an interdisciplinary chronic pain rehabilitation program from 1999-2005, 3) being on the waitlist to attend an interdisciplinary chronic pain rehabilitation program on September 1, 2012, or 4) being prescribed and dispensed any opioid medication used almost exclusively for pain (Table S1, Supplementary file 1) during the period from 1999-2011 as a subsidized patient of the NL Prescription Drug Program. b. The most performant Chronic Pain Algorithm.

Abbreviations: CPDM, Centre for Pain and Disability Management (an interdisciplinary chronic pain rehabilitation program); NLPDP, Newfoundland and Labrador Prescription Drug Plan (a financial assistance program covering eligible prescription medications to qualified seniors and low-income individuals/families); PC, encounter with anesthesiologist-recorded pain clinic Medical Care Plan provincial procedure billing code (Table S4, Supplementary file 1) in Medical Care Plan Fee-for-Service Physicians Claims File; P, encounter with physician-recorded pain-related diagnostic code (Table S3, Supplementary file 1) in Medical Care Plan Fee-for-Service Physicians Claims File; H, encounter with physician-recorded pain-related diagnostic code in Newfoundland and Labrador hospital Discharge Abstract Data; S, encounter with medical specialist-recorded pain-related diagnostic code (Table S3, Supplementary file 1) in Medical Care Plan Fee-for-Service Physicians Claims File or Newfoundland and Labrador hospital Discharge Abstract Data; YR, year(s).
