# Supplementary material for: Identifying cases of chronic pain using health administrative data: A validation study
Source: Can J Pain. 2020 Dec 3;4(1):252–67. doi: 10.1080/24740527.2020.1820857 (PMC7967902; doi:10.1080/24740527.2020.1820857)
Supplement: Supplemental Material [file UCJP_A_1820857_SM7355.zip › Supplementary file 3 changes accepted.docx]

**Table S6** Selection accuracy of chronic pain algorithms in Reference Standard Cohort^a^

| Algorithm | Selected  (n) | Test Prevalence  n/9715 | TP | FP | FN | TN | Sensitivity  (95% CI) | Specificity  (95% CI) | PPV  (95% CI) | NPV  (95% CI) | LR+  (95% CI) | LR-  (95% CI) | DOR  (95% CI) | Kappa  (95% CI) | aROC  (95% CI) |
| --- | --- | --- | --- | --- | --- | --- | --- | --- | --- | --- | --- | --- | --- | --- | --- |
| (1 PC ever) OR (2 P AND > 183 days between 2 P in 1 YR) | 2807 | 0.289 | 1066 | 1741 | 1320 | 5588 | 0.447  (0.427,0.467) | 0.762  (0.751,0.774) | 0.380  (0.362,0.398) | 0.809  (0.800,0.818) | 1.881  (1.770,1.998) | 0.726  (0.698,0.754) | 2.592  (2.348,2.856) | 0.197  (0.183,0.212) | 0.604  (0.591,0.618) |
| (1 PC ever) OR (2 P AND > 183 days between 2 P in 2 YR) | 4850 | 0.499 | 1704 | 3146 | 682 | 4183 | 0.714  (0.696,0.732) | 0.571  (0.560,0.581) | 0.351  (0.338,0.365) | 0.860  (0.850,0.870) | 1.664  (1.604,1.726) | 0.501  (0.469,0.535) | 3.322  (3.006,3.656) | 0.211  (0.198,0.225) | 0.642  (0.630,0.655) |
| (1 PC ever) OR (2 P AND > 183 days between 2 P n 3 YR) | 5820 | 0.599 | 1951 | 3869 | 435 | 3460 | 0.818  (0.802,0.833) | 0.472  (0.463,0.481) | 0.335  (0.323,0.347) | 0.888  (0.878,0.898) | 1.549  (1.505,1.594) | 0.386  (0.354,0.422) | 4.011  (3.580,4.493) | 0.195  (0.182,0.208) | 0.645  (0.633,0.657) |
| (1 PC ever) OR (2 P AND > 183 days between 2 P in 4 YR) | 6349 | 0.654 | 2060 | 4289 | 326 | 3040 | 0.863  (0.850,0.877) | 0.415  (0.407,0.423) | 0.324  (0.313,0.336) | 0.903  (0.893,0.913) | 1.475  (1.439,1.513) | 0.329  (0.297,0.366) | 4.479  (3.950,5.079) | 0.178  (0.165,0.191) | 0.639  (0.627,0.651) |
| (1 PC ever) OR (2 P AND > 183 days between 2 P in 5 YR) | 6703 | 0.690 | 2124 | 4579 | 262 | 2750 | 0.890  (0.878,0.903) | 0.375  (0.368,0.382) | 0.317  (0.306,0.328) | 0.913  (0.903,0.923) | 1.425  (1.393,1.457) | 0.293  (0.260,0.329) | 4.869  (4.246,5.582) | 0.165  (0.152,0.178) | 0.632  (0.621,0.644) |
| (1 PC ever) OR (2 P AND > 183 days between 2 P in 6 YR) | 6935 | 0.714 | 2167 | 4768 | 219 | 2561 | 0.908  (0.897,0.920) | 0.349  (0.343,0.356) | 0.312  (0.302,0.323) | 0.921  (0.911,0.931) | 1.396  (1.367,1.426) | 0.263  (0.231,0.299) | 5.315  (4.588,6.157) | 0.157  (0.144,0.170) | 0.629  (0.617,0.640) |
| (1 PC ever) OR (2 P AND > 183 days between 2 P in 7 YR) | 7083 | 0.729 | 2189 | 4894 | 197 | 2435 | 0.917  (0.906,0.928) | 0.332  (0.326,0.339) | 0.309  (0.298,0.320) | 0.925  (0.915,0.935) | 1.374  (1.347,1.402) | 0.249  (0.217,0.285) | 5.529  (4.741,6.447) | 0.150  (0.137,0.163) | 0.625  (0.612,0.636) |
| (1 PC ever) OR (3 P AND > 183 days between 2 P in 1 YR) | 2061 | 0.212 | 876 | 1185 | 1510 | 6144 | 0.367  (0.348,0.386) | 0.838  (0.827,0.849) | 0.425  (0.404,0.446) | 0.803  (0.794,0.812) | 2.271  (2.108,2.445) | 0.755  (0.731,0.780) | 3.008  (2.711,3.337) | 0.215  (0.200,0.230) | 0.603  (0.589,0.616) |
| (1 PC ever) OR (3 P AND > 183 days between 2 P in 2 YR) | 3753 | 0.386 | 1453 | 2300 | 933 | 5029 | 0.609  (0.589,0.629) | 0.686  (0.675,0.697) | 0.387  (0.372,0.403) | 0.844  (0.834,0.853) | 1.940  (1.852,2.033) | 0.570  (0.541,0.601) | 3.405  (3.094,3.748) | 0.247  (0.233,0.261) | 0.647  (0.634,0.660) |
| (1 PC ever) OR (3 P AND > 183 days between 2 P in 3 YR0 | 4720 | 0.486 | 1741 | 2979 | 645 | 4350 | 0.730  (0.712,0.747) | 0.594  (0.583,0.604) | 0.369  (0.355,0.383) | 0.871  (0.862,0.880) | 1.795  (1.730,1.863) | 0.455  (0.425,0.488) | 3.941  (3.560,4.363) | 0.243  (0.229,0.257) | 0.661  (0.649,0.674) |
| (1 PC ever) OR (3 P AND > 183 days between 2 P in 4 YR) | 5391 | 0.555 | 1912 | 3479 | 474 | 3850 | 0.801  (0.785,0.817) | 0.525  (0.516,0.534) | 0.355  (0.342,0.367) | 0.890  (0.881,0.900) | 1.688  (1.636,1.742) | 0.378  (0.348,0.411) | 4.464  (3.997,4.986) | 0.229  (0.216,0.243) | 0.663  (0.651,0.675) |
| (1 PC ever) OR (3 P AND > 183 days between 2 P in 5 YR) | 5821 | 0.599 | 2003 | 3818 | 383 | 3511 | 0.839  (0.825,0.854) | 0.479  (0.486,0.487) | 0.344  (0.332,0.356) | 0.902  (0.892,0.911) | 1.611  (1.567,1.657) | 0.335  (0.305,0.368) | 4.809  (4.259,5.414) | 0.214  (0.201,0.228) | 0.659  (0.647,0.671) |
| (1 PC ever) OR (3 P AND > 183 days between 2 P in 6 YR) | 6136 | 0.632 | 2079 | 4057 | 307 | 3272 | 0.871  (0.858,0.885) | 0.446  (0.439,0.454) | 0.339  (0.327,0.351) | 0.914  (0.905,0.923) | 1.574  (1.534,1.615) | 0.288  (0.259,0.321) | 5.462  (4.804,6.210) | 0.208  (0.195,0.221) | 0.659  (0.647,0.670) |
| (1 PC ever) OR (3 P AND > 183 days between 2 P in 7 YR) | 6339 | 0.652 | 2121 | 4218 | 265 | 3111 | 0.889  (0.876,0.902) | 0.424  (0.417,0.432) | 0.335  (0.323,0.346) | 0.922  (0.912,0.931) | 1.545  (1.508,1.582) | 0.262  (0.233,0.294) | 5.903  (5.153,6.762) | 0.201  (0.188,0.214) | 0.656  (0.645,0.668) |
| (1 PC ever) OR (4 P AND > 183 days between 2 P in 1 YR) | 1452 | 0.149 | 678 | 774 | 1708 | 6555 | 0.284  (0.266,0.302) | 0.894  (0.884,0.905) | 0.467  (0.441,0.493) | 0.793  (0.785,0.802) | 2.691  (2.454,2.950) | 0.800  (0.779,0.822) | 3.362  (2.994,3.775) | 0.206  (0.190,0.221) | 0.589  (0.575,0.603) |
| (1 PC ever) OR (4 P AND > 183 days between 2 P in 2 YR) | 2804 | 0.289 | 1186 | 1618 | 1200 | 5711 | 0.497  (0.477,0.517) | 0.779  (0.768,0.791) | 0.423  (0.405,0.441) | 0.826  (0.817,0.835) | 2.252  (2.123,2.388) | 0.645  (0.619,0.673) | 3.488  (3.165,3.845) | 0.261  (0.246,0.275) | 0.638  (0.625,0.651) |
| (1 PC ever) OR (4 P AND > 183 days between 2 P in 3 YR) | 3686 | 0.379 | 1494 | 2192 | 892 | 5137 | 0.626  (0.607,0.646) | 0.701  (0.690,0.712) | 0.405  (0.389,0.421) | 0.852  (0.843,0.861) | 2.094  (1.998,2.194) | 0.533  (0.505,0.563) | 3.925  (3.563,4.324) | 0.276  (0.262,0.290) | 0.663  (0.651,0.676) |
| (1 PC ever) OR (4 P AND > 183 days between 2 P in 4 YR) | 4397 | 0.453 | 1716 | 2681 | 670 | 4648 | 0.719  (0.701,0.737) | 0.634  (0.624,0.644) | 0.390  (0.376,0.405) | 0.874  (0.865,0.883) | 1.966  (1.890,2.045) | 0.443  (0.414,0.473) | 4.440  (4.013,4.913) | 0.275  (0.261,0.289) | 0.676  (0.664,0.689) |
| (1 PC ever) OR (4 P AND > 183 days between 2 P in 5 YR) | 4906 | 0.505 | 1850 | 3056 | 536 | 4273 | 0.775  (0.759,0.792) | 0.583  (0.573,0.593) | 0.377  (0.364,0.391) | 0.889  (0.880,0.897) | 1.859  (1.796,1.925) | 0.385  (0.357,0.416) | 4.826  (4.337,5.370) | 0.264  (0.251,0.278) | 0.679  (0.667,0.691) |
| (1 PC ever) OR (4 P AND > 183 days between 2 P in 6 YR) | 5287 | 0.544 | 1939 | 3348 | 447 | 3981 | 0.813  (0.797,0.828) | 0.543  (0.534,0.552) | 0.367  (0.354,0.380) | 0.899  (0.890,0.908) | 1.779  (1.724,1.836) | 0.345  (0.316,0.376) | 5.158  (4.608,5.753) | 0.252  (0.239,0.266) | 0.678  (0.666,0.689) |
| (1 PC ever) OR (4 P AND > 183 days between 2 P in 7 YR) | 5554 | 0.572 | 2009 | 3545 | 377 | 3784 | 0.842  (0.827,0.857) | 0.516  (0.508,0.525) | 0.362  (0.349,0.374) | 0.909  (0.901,0.918) | 1.741  (1.690,1.793) | 0.306  (0.278,0.337) | 5.688  (5.049,6.408) | 0.247  (0.234,0.261) | 0.679  (0.667,0.691) |
| (1 PC ever) OR (5 P AND > 183 days between 2 P in 1 YR) | 1057 | 0.109 | 521 | 536 | 1865 | 6793 | 0.218  (0.202,0.235) | 0.927  (0.917,0.936) | 0.493  (0.463,0.523) | 0.785  (0.776,0.793) | 2.986  (2.671,3.337) | 0.843  (0.825,0.862) | 3.540  (3.106,4.036) | 0.179  (0.163.0.194) | 0.572  (0.559,0.586) |
| (1 PC ever) OR (5 P AND > 183 days between 2 P in 2 YR) | 2118 | 0.218 | 968 | 1150 | 1418 | 6179 | 0.406  (0.386,0.425) | 0.843  (0.832,0.854) | 0.457  (0.436,0.478) | 0.813  (0.805,0.822) | 2.586  (2.406,2.778) | 0.705  (0.681,0.730) | 3.668  (3.308,4.066) | 0.259  (0.244,0.273) | 0.624  (0.611,0.638) |
| (1 PC ever) OR (5 P AND > 183 days between 2 P in 3 YR) | 2902 | 0.299 | 1252 | 1650 | 1134 | 5679 | 0.525  (0.505,0.545) | 0.775  (0.763,0.786) | 0.431  (0.413,0.449) | 0.834  (0.825,0.842) | 2.331  (2.201,2.468) | 0.613  (0.587,0.641) | 3.800  (3.448,4.188) | 0.279  (0.265,0.293) | 0.650  (0.636,0.663) |
| (1 PC ever) OR (5 P AND > 183 days between 2 P in 4 YR) | 3555 | 0.366 | 1483 | 2072 | 903 | 5257 | 0.622  (0.602,0.641) | 0.717  (0.706,0.728) | 0.417  (0.401,0.433) | 0.853  (0.845,0.862) | 2.198  (2.095,2.307) | 0.528  (0.500,0.557) | 4.167  (3.781,4.592) | 0.291  (0.277,0.305) | 0.669  (0.656,0.682) |
| *(1 PC ever) OR (5 P AND > 183 days between 2 P in 5 YR)^b^* | ***4113*** | ***0.423*** | ***1678*** | ***2435*** | ***708*** | ***4894*** | ***0.703***  ***(0.685,0.722)*** | ***0.668***  ***(0.657,0.678)*** | ***0.408***  ***(0.393,0.423)*** | ***0.874***  ***(0.865,0.882)*** | ***2.117***  ***(2.030,2.207)*** | ***0.444***  ***(0.417,0.474)*** | ***4.763***  ***(4.308,5.267)*** | ***0.298***  ***(0.285,0.312)*** | ***0.685***  ***(0.673,0.698)*** |
| (1 PC ever) OR (5 P AND > 183 days between 2 P in 6 YR) | 4553 | 0.469 | 1794 | 2759 | 592 | 4570 | 0.752  (0.735,0.769) | 0.624  (0.614,0.633) | 0.394  (0.380,0.408) | 0.885  (0.877,0.894) | 1.997  (1.924,2.073) | 0.398  (0.370,0.428) | 5.020  (4.523,5.571) | 0.287  (0.274,0.301) | 0.688  (0.676,0.700) |
| (1 PC ever) OR (5 P AND > 183 days between 2 P in 7 YR) | 4881 | 0.502 | 1883 | 2998 | 503 | 4331 | 0.789  (0.773,0.806) | 0.591  (0.582,0.600) | 0.386  (0.372,0.399) | 0.896  (0.887,0.905) | 1.929  (1.864,1.997) | 0.357  (0.329,0.386) | 5.408  (4.850,6.030) | 0.281  (0.268,0.295) | 0.690  (0.678,0.702) |
| (1 PC ever) OR ((2 P AND > 183 days between 2 P in 1 YR) AND >/= 1 S ever) | 2089 | 0.215 | 887 | 1202 | 1499 | 6127 | 0.372  (0.352,0.391) | 0.836  (0.825,0.847) | 0.425  (0.403,0.446) | 0.803  (0.795,0.812) | 2.267  (2.106,2.439) | 0.751  (0.727,0.776) | 3.016  (2.720,3.345) | 0.217  (0.202,0.232) | 0.604  (0.590,0.617) |
| (1 PC ever) OR ((2 P AND > 183 days between 2 P in 2 YR) AND >/= 1 S ever) | 3360 | 0.346 | 1343 | 2017 | 1043 | 5312 | 0.563  (0.543,0.583) | 0.725  (0.713,0.736) | 0.400  (0.383,0.416) | 0.836  (0.827,0.845) | 2.045  (1.943,2.153) | 0.603  (0.575,0.633) | 3.391  (3.081,3.732) | 0.253  (0.239,0.267) | 0.644  (0.631,0.657) |
| (1 PC ever) OR ((2 P AND > 183 days between 2 P in 3 YR) AND >/= 1 S ever) | 3920 | 0.403 | 1517 | 2403 | 869 | 4926 | 0.636  (0.616,0.655) | 0.672  (0.661,0.683) | 0.387  (0.372,0.402) | 0.850  (0.841,0.859) | 1.939  (1.854,2.028) | 0.542  (0.513,0.573) | 3.579  (3.249,3.941) | 0.253  (0.239,0.267) | 0.654  (0.641,0.667) |
| (1 PC ever) OR ((2 P AND > 183 days between 2 P in 4 YR) AND >/= 1 S ever) | 4190 | 0.431 | 1585 | 2605 | 801 | 4724 | 0.664  (0.645,0.683) | 0.645  (0.634,0.655) | 0.378  (0.364,0.393) | 0.855  (0.846,0.864) | 1.869  (1.792,1.949) | 0.521  (0.491,0.552) | 3.588  (3.255,3.956) | 0.246  (0.232,0.260) | 0.654  (0.642,0.667) |
| (1 PC ever) OR ((2 P AND > 183 days between 2 P in 5 YR) AND >/= 1 S ever) | 4388 | 0.452 | 1628 | 2760 | 758 | 4569 | 0.682  (0.664,0.701) | 0.623  (0.613,0.634) | 0.371  (0.357,0.385) | 0.858  (0.848,0.867) | 1.812  (1.740,1.886) | 0.510  (0.479,0.542) | 3.555  (3.223,3.923) | 0.238  (0.224,0.252) | 0.653  (0.640,0.665) |
| (1 PC ever) OR ((2 P AND > 183 days between 2 P in 6 YR) AND >/= 1 S ever) | 4508 | 0.464 | 1654 | 2854 | 732 | 4475 | 0.693  (0.675,0.712) | 0.611  (0.600,0.621) | 0.367  (0.353,0.381) | 0.859  (0.850,0.869) | 1.780  (1.712,1.851) | 0.502  (0.472,0.535) | 3.543  (3.209,3.911) | 0.234  (0.220,0.248) | 0.652  (0.639,0.664) |
| (1 PC ever) OR ((2 P AND > 183 days between 2 P in 7 YR) AND >/= 1 S ever) | 4579 | 0.471 | 1666 | 2913 | 720 | 4416 | 0.698  (0.680,0.717) | 0.603  (0.592,0.613) | 0.364  (0.350,0.378) | 0.860  (0.850,0.869) | 1.757  (1.690,1.826) | 0.501  (0.470,0.534) | 3.508  (3.177,3.873) | 0.230  (0.216,0.243) | 0.650  (0.638,0.663) |
| (1 PC ever) OR ((3 P AND > 183 days between 2 P in 1 YR) AND >/= 1 S ever) | 1645 | 0.169 | 753 | 892 | 1633 | 6437 | 0.316  (0.297,0.334) | 0.878  (0.868,0.889) | 0.458  (0.434,0.482) | 0.798  (0.789,0.806) | 2.593  (2.381,2.824) | 0.779  (0.757,0.802) | 3.328  (2.977,3.719) | 0.217  (0.201,0.232) | 0.597  (0.583,0.611) |
| (1 PC ever) OR ((3 P AND > 183 days between 2 P in 2 YR) AND >/= 1 S ever) | 2812 | 0.289 | 1186 | 1626 | 1200 | 5703 | 0.497  (0.477,0.517) | 0.778  (0.767,0.790) | 0.422  (0.404,0.440) | 0.826  (0.817,0.835) | 2.240  (2.112,2.376) | 0.646  (0.620,0.674) | 3.466  (3.145,3.821) | 0.260  (0.245,0.274) | 0.637  (0.624,0.651) |
| (1 PC ever) OR ((3 P AND > 183 days between 2 P in 3 YR) AND >/= 1 S ever) | 3417 | 0.352 | 1399 | 2018 | 987 | 5311 | 0.586  (0.567,0.606) | 0.725  (0.713,0.736) | 0.409  (0.393,0.426) | 0.843  (0.834,0.852) | 2.129  (2.025,2.239) | 0.571  (0.543,0.600) | 3.730  (3.388,4.107) | 0.271  (0.256,0,286) | 0.655  (0.642,0.668) |
| (1 PC ever) OR ((3 P AND > 183 days between 2 P in 4 YR) AND >/= 1 S ever) | 3810 | 0.392 | 1509 | 2301 | 877 | 5028 | 0.632  (0.613,0.652) | 0.686  (0.675,0.697) | 0.396  (0.381,0.412) | 0.851  (0.842,0.861) | 2.014  (1.925,2.108) | 0.536  (0.507,0.566) | 3.760  (3.413,4.142) | 0.265  (0.251,0.279) | 0.659  (0.646,0.672) |
| (1 PC ever) OR ((3 P AND > 183 days between 2 P in 5 YR) AND >/= 1 S ever) | 4047 | 0.417 | 1566 | 2481 | 820 | 4848 | 0.656  (0.637,0.675) | 0.661  (0.651,0.672) | 0.387  (0.372,0.402) | 0.855  (0.846,0.864) | 1.939  (1.857,2.024) | 0.520  (0.490,0.550) | 3.732  (3.386,4.113) | 0.257  (0.243,0.271) | 0.659  (0.646,0.671) |
| (1 PC ever) OR ((3 P AND > 183 days between 2 P in 6 YR) AND >/= 1 S ever) | 4225 | 0.435 | 1613 | 2612 | 773 | 4717 | 0.676  (0.657,0.695) | 0.644  (0.633,0.654) | 0.382  (0.367,0.396) | 0.859  (0.850,0.868) | 1.897  (1.820,1.977) | 0.503  (0.474,0.535) | 3.768  (3.416,4.157) | 0.254  (0.235,0.268) | 0.660  (0.647,0.672) |
| (1 PC ever) OR ((3 P AND > 183 days between 2 P in 7 YR) AND >/= 1 S ever) | 4331 | 0.446 | 1636 | 2695 | 750 | 4634 | 0.686  (0.667,0.704) | 0.632  (0.622,0.643) | 0.378  (0.363,0.392) | 0.861  (0.851,0.870) | 1.865  (1.791,1.942) | 0.497  (0.467,0.529) | 3.751  (3.399,4.139) | 0.249  (0.236,0.263) | 0.659  (0.646,0.671) |
| (1 PC ever) OR ((4 P AND > 183 days between 2 P in 1 YR) AND >/= 1 S ever) | 1231 | 0.127 | 603 | 628 | 1783 | 6701 | 0.253  (0.235,0.270) | 0.914  (0.904,0.924) | 0.490  (0.462,0.518) | 0.790  (0.781,0.799) | 2.949  (2.664,3.265) | 0.817  (0.798,0.837) | 3.609  (3.190,4.082) | 0.200  (0.184,0.215) | 0.583  (0.569,0.597) |
| (1 PC ever) OR ((4 P AND > 183 days between 2 P in 2 YR) AND >/= 1 S ever) | 2253 | 0.232 | 1012 | 1241 | 1374 | 6088 | 0.424  (0.404,0.444) | 0.831  (0.819,0.842) | 0.449  (0.429,0.470) | 0.816  (0.807,0.825) | 2.505  (2.338,2.684) | 0.693  (0.669,0.719) | 3.613  (3.264,4.000) | 0.260  (0.245,0.274) | 0.627  (0.614,0.641) |
| (1 PC ever) OR ((4 P AND > 183 days between 2 P in 3 YR) AND >/= 1 S ever) | 2857 | 0.294 | 1248 | 1608 | 1138 | 5721 | 0.523  (0.503,0.543) | 0.781  (0.769,0.792) | 0.437  (0.419,0.455) | 0.834  (0.825,0.843) | 2.384  (2.250,2.526) | 0.611  (0.585,0.638) | 3.902  (3.539,4.302) | 0.285  (0.270,0.299) | 0.652  (0.638,0.665) |
| (1 PC ever) OR ((4 P AND > 183 days between 2 P in 4 YR) AND >/= 1 S ever) | 3307 | 0.340 | 1400 | 1907 | 986 | 5422 | 0.587  (0.567,0.607) | 0.740  (0.729,0.751) | 0.423  (0.407,0.440) | 0.846  (0.837,0.855) | 2.255  (2.142,2.374) | 0.559  (0.532,0.587) | 4.037  (3.665,4.447) | 0.289  (0.275,0.303) | 0.663  (0.650,0.676) |
| (1 PC ever) OR ((4 P AND > 183 days between 2 P in 5 YR) AND >/= 1 S ever) | 3622 | 0.373 | 1490 | 2132 | 896 | 5197 | 0.624  (0.605,0.644) | 0.709  (0.698,0.720) | 0.411  (0.395,0.427) | 0.853  (0.844,0.862) | 2.147  (2.047,2.251) | 0.530  (0.502,0.559) | 4.054  (3.679,4.466) | 0.284  (0.270,0.298) | 0.667  (0.654,0.679) |
| (1 PC ever) OR ((4 P AND > 183 days between 2 P in 6 YR) AND >/= 1 S ever) | 3847 | 0.396 | 1548 | 2299 | 838 | 5030 | 0.649  (0.630,0.668) | 0.686  (0.675,0.697) | 0.402  (0.387,0.418) | 0.857  (0.848,0.866) | 2.068  (1.977,2.163) | 0.512  (0.484,0.542) | 4.042  (3.666,4.455) | 0.278  (0.264,0.292) | 0.667  (0.655,0.680) |
| (1 PC ever) OR ((4 P AND > 183 days between 2 P in 7 YR) AND >/= 1 S ever) | 4005 | 0.412 | 1588 | 2417 | 798 | 4912 | 0.666  (0.647,0.684) | 0.670  (0.659,0.681) | 0.397  (0.381,0.412) | 0.860  (0.851,0.869) | 2.018  (1.933,2.107) | 0.499  (0.471,0.529) | 4.044  (3.667,4.462) | 0.273  (0.259,0.287) | 0.668  (0.655,0.680) |
| (1 PC ever) OR ((5 P AND > 183 days between 2 P in 1 YR) AND >/= 1 S ever) | 931 | 0.096 | 478 | 453 | 1908 | 6876 | 0.200  (0.184,0.216) | 0.938  (0.929,0.947) | 0.513  (0.481,0.546) | 0.783  (0.774,0.791) | 3.241  (2.875,3.654) | 0.852  (0.835,0.870) | 3.803  (3.312,4.366) | 0.174  (0.159,0.190) | 0.569  (0.555,0.583) |
| (1 PC ever) OR ((5 P AND > 183 days between 2 P in 2 YR) AND >/= 1 S ever) | 1787 | 0.184 | 849 | 938 | 1537 | 6391 | 0.356  (0.337,0.375) | 0.872  (0.861,0.883) | 0.475  (0.452,0.498) | 0.806  (0.797,0.815) | 2.780  (2.565,3.013) | 0.739  (0.716,0.762) | 3.764  (3.377,4.194) | 0.249  (0.234,0.264) | 0.614  (0.600,0.627) |
| (1 PC ever) OR ((5 P AND > 183 days between 2 P in 3 YR) AND >/= 1 S ever) | 2356 | 0.243 | 1073 | 1283 | 1313 | 6046 | 0.450  (0.430,0.470) | 0.825  (0.814,0.836) | 0.455  (0.435,0.476) | 0.822  (0.813,0.830) | 2.569  (2.403,2.746) | 0.667  (0.642,0.693) | 3.851  (3.482,4.259) | 0.276  (0.261,0.290) | 0.637  (0.624,0.651) |
| (1 PC ever) OR ((5 P AND > 183 days between 2 P in 4 YR) AND >/= 1 S ever) | 2815 | 0.290 | 1250 | 1565 | 1136 | 5764 | 0.524  (0.504,0.544) | 0.786  (0.775,0.798) | 0.444  (0.426,0.462) | 0.835  (0.827,0.844) | 2.453  (2.315,2.601) | 0.605  (0.579,0.632) | 4.053  (3.675,4.469) | 0.293  (0.278,0.307) | 0.655  (0.642,0.668) |
| (1 PC ever) OR ((5 P AND > 183 days between 2 P in 5 YR) AND >/= 1 S ever) | 3183 | 0.328 | 1389 | 1794 | 997 | 5535 | 0.582  (0.562,0.602) | 0.755  (0.744,0.767) | 0.436  (0.419,0.454) | 0.847  (0.839,0.856) | 2.378  (2.256,2.507) | 0.553  (0.527,0.581) | 4.298  (3.900,4.737) | 0.303  (0.289,0.317) | 0.668  (0.656,0.681) |
| (1 PC ever) OR ((5 P AND > 183 days between 2 P in 6 YR) AND >/= 1 S ever) | 3457 | 0.356 | 1462 | 1994 | 923 | 5335 | 0.613  (0.594,0.633) | 0.728  (0.717,0.739) | 0.423  (0.407,0.440) | 0.853  (0.844,0.861) | 2.254  (2.146,2.367) | 0.531  (0.504,0.560) | 4.241  (3.848,4.673) | 0.296  (0.282,0.310) | 0.670  (0.658,0.683) |
| PC ever) OR ((5 P AND > 183 days between 2 P in 7 YR) AND >/= 1 S ever) | 3665 | 0.377 | 1519 | 2146 | 867 | 5183 | 0.637  (0.617,0.656) | 0.707  (0.696,0.718) | 0.414  (0.399,0.430) | 0.857  (0.848,0.866) | 2.174  (2.075,2.278) | 0.514  (0.486,0.543) | 4.231  (3.839,4.664) | 0.291  (0.277,0.305) | 0.672  (0.659,0.684) |

Notes: a. The Reference Standard Cohort was comprised of primary care patients of all ages who met the inclusion criteria of implied consent to participate in the Canadian Primary Care Sentinel Surveillance Network-Newfoundland and Labrador since December 31, 2009 or earlier and had a minimum of two years of electronic medical record data for analysis. b. The most performant Chronic Pain Algorithm.

Abbreviations: TP, true positive; FP, false positive; FN, false negative; TN, true negative; CI, confidence interval; PPV, positive predictive value; NPV, negative predictive value; LR+, likelihood ratio positive; LR-, likelihood ratio negative; DOR, diagnostic odds ratio; aROC, area under the Receiver Operating Characteristic curve; PC, encounter with anesthesiologist-recorded pain clinic Medical Care Plan provincial procedure billing code (Table S4, Supplementary file 1) in Medical Care Plan Fee-for-Service Physicians Claims File; P, encounter with physician-recorded pain-related diagnostic code (Table S3, Supplementary file 1) in Medical Care Plan Fee-for-Service Physicians Claims File; YR, year(s); S, encounter with medical specialist-recorded pain-related diagnostic code (Table S3, Supplementary file 1) in Medical Care Plan Fee-for-Service Physicians Claims File or Newfoundland and Labrador Provincial hospital Discharge Abstract Data.
