# Supplementary material for: Identifying cases of chronic pain using health administrative data: A validation study
Source: Can J Pain. 2020 Dec 3;4(1):252–67. doi: 10.1080/24740527.2020.1820857 (PMC7967902; doi:10.1080/24740527.2020.1820857)
Supplement: Supplemental Material [file UCJP_A_1820857_SM7355.zip › Supplementary file 4, Oct 8, 2020.docx]

**Supplementary materials:** Figure S1 Chronic pain algorithm sensitivity versus specificity plot from validation step

Table S7 Five most common and five least common ICD-9 pain-related codes used for Chronic Pain Algorithm selection


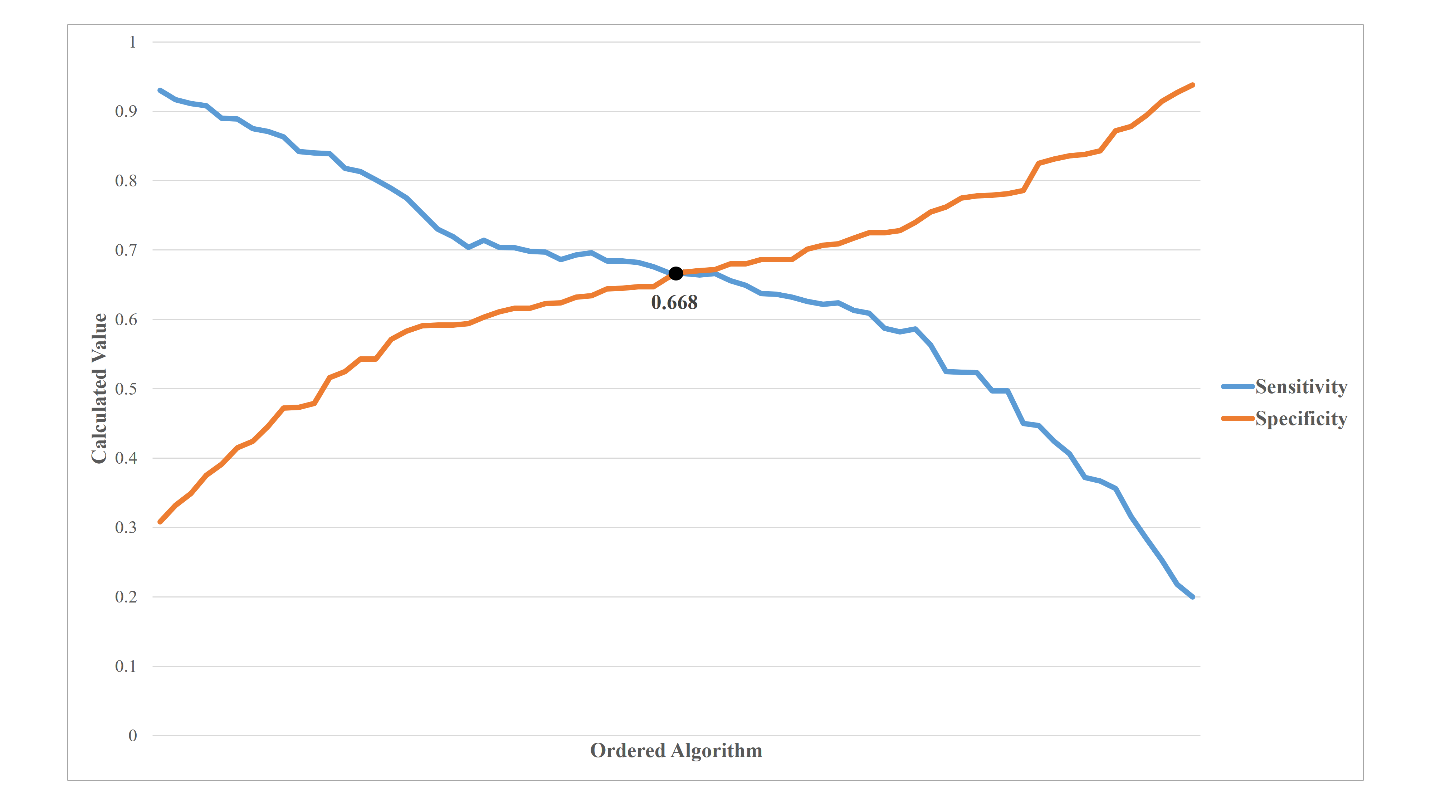


Figure S1. Chronic pain algorithm sensitivity versus specificity plot from validation step.

Note: The 56 chronic pain administrative data algorithms tested against the reference standard in the Reference Standard Cohort from the final algorithm validation and selection step were ordered to determine where the calculated sensitivity and specificity plot values intersected.

**Table S7.** Five most common and five least common ICD-9 pain-related codes used for Chronic Pain Algorithm^a^ selection.

| **Most common ICD-9 pain-related diagnostic codes** | | **Least common ICD-9 pain-related diagnostic codes** | |
| --- | --- | --- | --- |
| **ICD-9 Code** | **Description** | **ICD-9 Code** | **Description** |
| 724 | Other and unspecified disorders of back | 738 | Other acquired deformity |
| 781 | Symptoms Involving nervous and musculoskeletal systems | 756 | Other congenital musculoskeletal anomalies |
| 715 | Osteoarthritis and allied disorders | 831 | Dislocation of shoulder |
| 564 | Functional digestive disorders, not elsewhere classified | 846 | Sprains and strains of sacroiliac region |
| 714 | Rheumatoid arthritis and other inflammatory polyarthopathies | 843 | Sprains and strains of hip and thigh |

Notes: a. Chronic Pain Algorithm used to identify cases of chronic pain from the Medical Care Plan Physician Fee-for-Service Claims File in Newfoundland and Labrador defined as: 1) a single encounter date with an anesthesiologist recording a chronic pain-related provincial Medical Care Plan procedure billing code (Table S4, Supplementary file 1) in the Medical Care Plan Fee-for-Service Physicians Claims File; OR 2) five or more encounter dates with a physician recording any pain-related diagnostic code (Table S3, Supplementary file 1) in a five-year period with more than 183 days separating at least two pain-related encounter dates in the Medical Care Plan Fee-for-Service Physicians Claims File.

Abbreviations: ICD-9, *International Classification of Disease – 9^th^ Revision*
