# Supplementary figures and images for: Identifying cases of chronic pain using health administrative data: A validation study
Source: Can J Pain. 2020 Dec 3;4(1):252–67. doi: 10.1080/24740527.2020.1820857 (PMC7967902; doi:10.1080/24740527.2020.1820857)

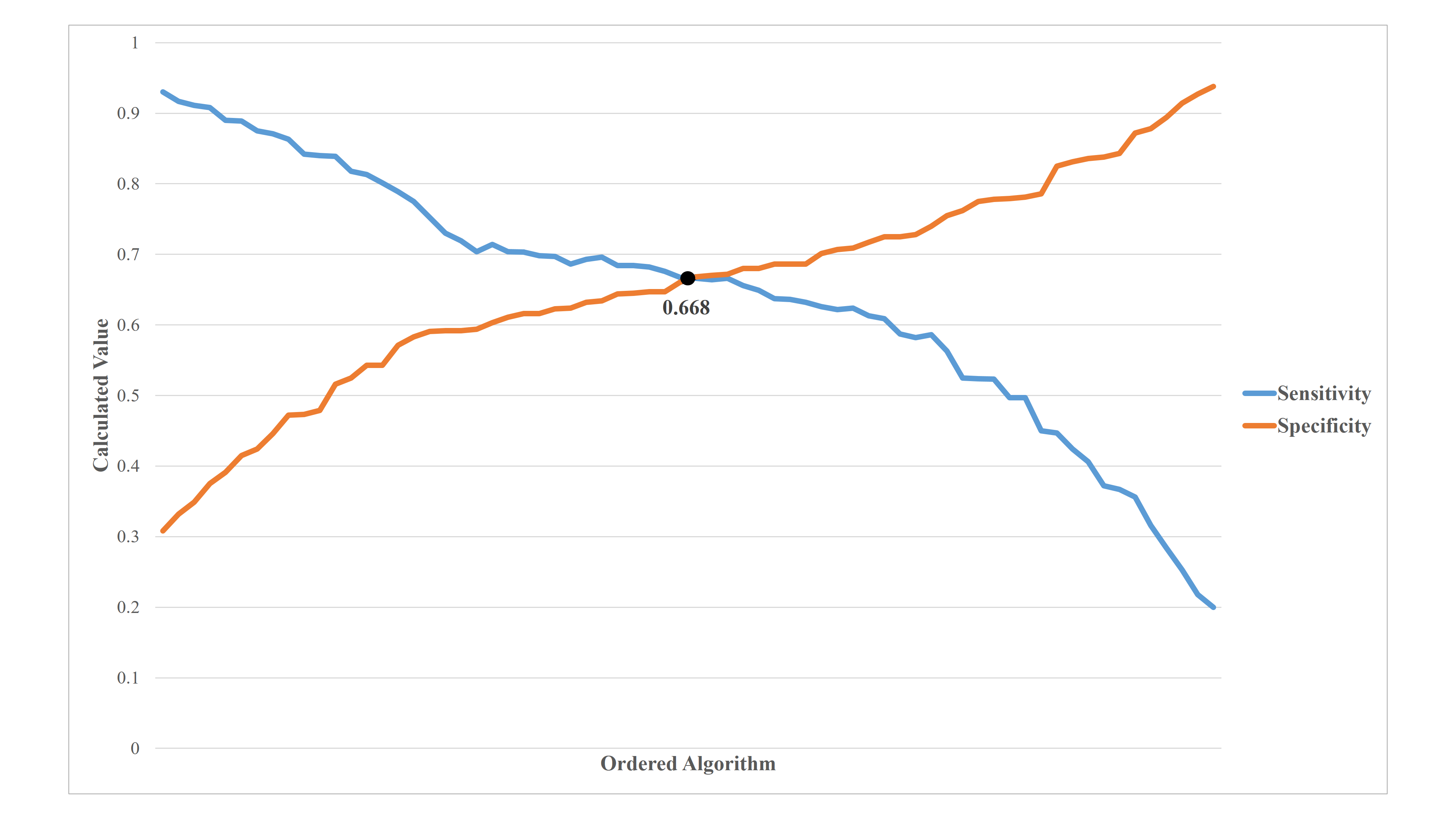

Supplement: Supplemental Material [file UCJP_A_1820857_SM7355.zip › Figure S1, Oct 8, 2020.TIF]
